# Endogenous Near‐Infrared Chemiluminescence: Imaging‐Guided Non‐Invasive Thrombolysis and Anti‐Inflammation Based on a Heteronuclear Transition Metal Complex

**DOI:** 10.1002/advs.202501257

**Published:** 2025-03-26

**Authors:** Ziwei Wang, Bo Zhu, Wenxin Nie, Liping Zhang, Nan Xiao, Qiaohua Zhang, Zihan Wu, Chunguang Shi, Weijin Zhu, Qianwen Liu, Dongxia Zhu, Martin R. Bryce, Lijie Ren, Ben Zhong Tang

**Affiliations:** ^1^ Key Laboratory of Nanobiosensing and Nanobioanalysis at Universities of Jilin Province Department of Chemistry Northeast Normal University 5268 Renmin Street Changchun Jilin 130024 P. R. China; ^2^ Department of Neurology Inst Translat Med The First Affiliated Hospital of Shenzhen University Shenzhen Second People's Hospital Shenzhen 518035 P. R. China; ^3^ Department of Chemistry Durham University Durham DH1 3LE UK; ^4^ Department of Chemistry Hong Kong Branch of Chinese National Engineering Research Center for Tissue Restoration and Reconstruction State Key Laboratory of Molecular Neuroscience Division of Life Science Ming Wai Lau Centre for Reparative Medicine Karolinska Institute and Guangdong‐Hong Kong‐Macau Joint Laboratory of Optoelectronic and Magnetic Functional Materials The Hong Kong University of Science and Technology Kowloon Hong Kong 999077 P. R. China; ^5^ School of Science and Engineering Shenzhen Institute of Aggregate Science and Technology The Chinese University of Hong Kong Shenzhen Guangdong 518172 P. R. China

**Keywords:** Chemiluminescence Image, Iridium complex, Porphyrin derivatives, photothermal therapy, photodynamic therapy

## Abstract

Conventional therapy to treat thrombi (blood clots) has significant limitations: i) inflammation; ii) bleeding side effects; iii) re‐embolisation, and iv) in situ thrombi that are not visible. Here it is reported that Cu2Ir nanoparticles (NPs) with a Cu‐coordinated tetraphenylporphyrin (TPP) core and cyclometalated Ir(C^N)_2_(N^N) substituents integrate long‐lived near‐infrared (NIR) chemiluminescence (CL) imaging, photothermal therapy (PTT) and photodynamic therapy (PDT) for thrombolysis, with antioxidant and anti‐inflammatory properties. Based on density functional theory calculations the chemiluminescent reaction site between TPP and peroxynitrite (ONOO^−^) is confirmed for the first time. The presence of the transition metal significantly improves the chemiluminescent properties of TPP. Upon specific activation by ONOO^−^, Cu2Ir NPs exhibited more than 30‐fold NIR CL intensity than TPP NPs, and the luminescence lasted for 60 min allowing for precise and long‐lasting dynamic tracking of thrombi. Cu2Ir NPs achieved non‐invasive safe thrombolytic therapy triggered by NIR irradiation at the signaling site. 72.3% blood reperfusion is obtained for nearly complete restoration of blood flow, and re‐embolism is prevented in a mouse carotid artery model. Furthermore, Cu2Ir NPs scavenged excess reactive oxygen/nitrogen species (RONS) and reduced inflammatory factors. Cu2Ir NPs hold promise as a single‐molecule strategy for diagnosing and treating diseases associated with thrombosis.

## Graphical abstract

Cu2Ir NPs based on a Cu‐porphyrin core and cyclometalated Ir(III) substituents achieve combined endogenous peroxynitrite‐triggered NIR chemiluminescence imaging, PTT and PDT for successfully achieving safe thrombolysis, antioxidant and anti‐inflammatory multifunctionality in one single molecule.

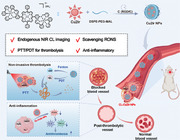

## 1 Introduction

Thrombotic and embolic cardiovascular diseases are major causes of morbidity and mortality worldwide.^[^ ^1^ ^]^ Currently, the clinically used antithrombotic drugs are urokinase (UK) which is a serine protease, and the tissue‐type plasminogen activator (rt‐PA), which dislodge a thrombus (blood clot) and recanalize the blocked vessel.^[^ ^2^ ^]^ However, drug therapies usually cause complications with bleeding, and have the disadvantages of short half‐lives, high drug dosage, and low selectivity.^[^ ^3^ ^]^ Moreover, thrombosis and thrombolytic drug‐induced vascular endothelial injuries activate platelets, leading to the generation of excess reactive oxygen/nitrogen species (RONS) and a significant increase in oxidative stress.^[^ ^4^ ^]^ Subsequently, this stress can stimulate the overexpression of inflammatory factors in vascular endothelial cells, promoting increased platelet activation. This creates a vicious circle that ultimately worsens vascular conditions and elevates the risk of thrombotic complications.^[^ ^5^ ^]^ Therefore, scavenging excess RONS and inhibiting inflammatory factors during thrombolysis are highly desirable to improve antithrombotic efficacy and biosafety.^[^ ^6^ ^]^

Non‐pharmacological therapies, including photothermal therapy (PTT) and photodynamic therapy (PDT) have great antithrombotic potential with the advantages of spatial‐temporal precision, high intrinsic specificity, and minimal invasiveness when compared with conventional treatments.^[^ ^7^ ^]^ PTT thrombolysis involves the local generation of hyperthermia to achieve thermal ablation of thrombus clots. Regrettably, the fibrin skeleton within the thrombus cannot be destroyed using PTT which generally causes secondary embolism.^[^ ^8^ ^]^ Gratifyingly, reports have confirmed that singlet oxygen (^1^O_2_) generated by PDT and hydroxyl radicals (•OH) produced by the Fenton reaction can effectively break down the fibrin backbone, addressing the limitations in thrombolysis and the risk of thrombotic recurrence following PTT.^[^ ^9^ ^]^ Recently, dual PDT/PTT photosensitizers (PSs) have shown combined advantages to achieve complete thrombolysis and to prevent secondary embolism from post‐photothermal fragments.^[^ ^10^ ^]^ In our previous work Ir(III) complex PSs with excellent PDT/PTT properties achieved significant therapeutic outcomes for tumour therapy.^[^ ^11^ ^]^ Nonetheless, excess RONS generated during phototherapy can disrupt cell membrane structures, causing damage to neighbouring tissues and leading to poor prognosis; these drawbacks are frequently overlooked.^[^ ^12^ ^]^ Thus, how to achieve synergistic PDT/PTT thrombolysis while avoiding inflammation caused by excessive RONS production is crucial and highly challenging.

Scavenging RONS by utilizing broad‐spectrum antioxidants, such as *N*‐acetylcysteine (NAC) and monomethyl fumarate (MMF) is a promising strategy for the treatment of various inflammatory disorders.^[^ ^13^ ^]^ However, owing to their poor stability, unsatisfactory efficacy and inadequate bioavailability, these drugs still fail to achieve the ideal therapeutic effects for RONS‐related diseases in clinical practice.^[^ ^14^ ^]^ Recent studies have verified that transition metal nanozymes can exhibit superoxide dismutase (SOD)‐like and catalase (CAT)‐like activities through reversible switching of valence states, and then enabling the clearance of RONS.^[^ ^15^ ^]^ For example, Liu et al. obtained ultra‐small Cu_5.4_O NPs exhibiting multiple enzyme‐mimicking and broad‐spectrum RONS scavenging ability for the treatment of acute liver injury and wound healing.^[^ ^16^ ^]^ Guo et al. created manganese‐based biomimetic nanoenzymes (MF@S) that reduce RONS leakage and accumulation through the reduction of inflammatory factor secretion to remodel the diabetic microenvironment.^[^ ^17^ ^]^ These nanozymes can scavenge cytotoxic free radicals and exhibit higher stability than natural enzymes.^[^ ^18^ ^]^ Unfortunately, multiple components and complex construction of nanozymes has impeded their clinical application. In contrast, molecular complexes are not only chemically simple and well‐defined, but they can also simultaneously integrate multiple functions in a single molecule through rational design.^[^ ^19^ ^]^ Consequently, further research on transition metal complexes is vital to provide new insights and guidance for the effective clinical application of multifunctional thrombolytic drugs.

Phototheranostics guided by imaging is now a standard approach for accurate diagnosis and effective treatment of a wide range of diseases.^[^ ^20^ ^]^ In this context, fluorescence imaging has been widely studied, but it depends on excitation light which usually has limited penetration depth in tissue. Meanwhile the natural autofluorescence of biological tissues make imaging less than ideal.^[^ ^21^ ^]^ Different from fluorescence imaging, chemiluminescence (CL) is triggered by a chemical reaction without a light source, thereby avoiding photodamage to tissues caused by strong excitation light.^[^ ^22^ ^]^ Nonetheless, chemiluminescent PSs suffer from short‐wavelength emission, complex synthesis, and poor stability, which limits their clinical application, especially in treating deep‐seated diseases.^[^ ^23^ ^]^ For long‐wavelength emission, metal‐free porphyrin derivatives exhibit CL properties. For example, vinylidene bonds of pyropheophorbide A (Ppa) are successively oxidized upon interaction with peroxynitrite (ONOO^−^) to produce persistent luminescence in the NIR region (>700 nm).^[^ ^24^ ^]^ However, porphyrins usually suffer from poor water solubility, limited chemical stability and low bio‐stability.^[^ ^25^ ^]^ The coordination of metal atoms into porphyrins can effectively enhance the stability of the ring, especially some highly charged (+2 and +3) transition metal cations show significant stability in their metal‐N_4_ coordination bonds and can only be demetallized by dismantling the macrocycle.^[^ ^26^ ^]^ The transition metals within the macrocycle also favor reactions with substances of different electronegativity.^[^ ^27^ ^]^ We postulated that this particular interaction will provide important theoretical and practical implications for the rational construction of metalloporphyrin complexes to realize strong NIR chemiluminescence.

**Scheme 1:**
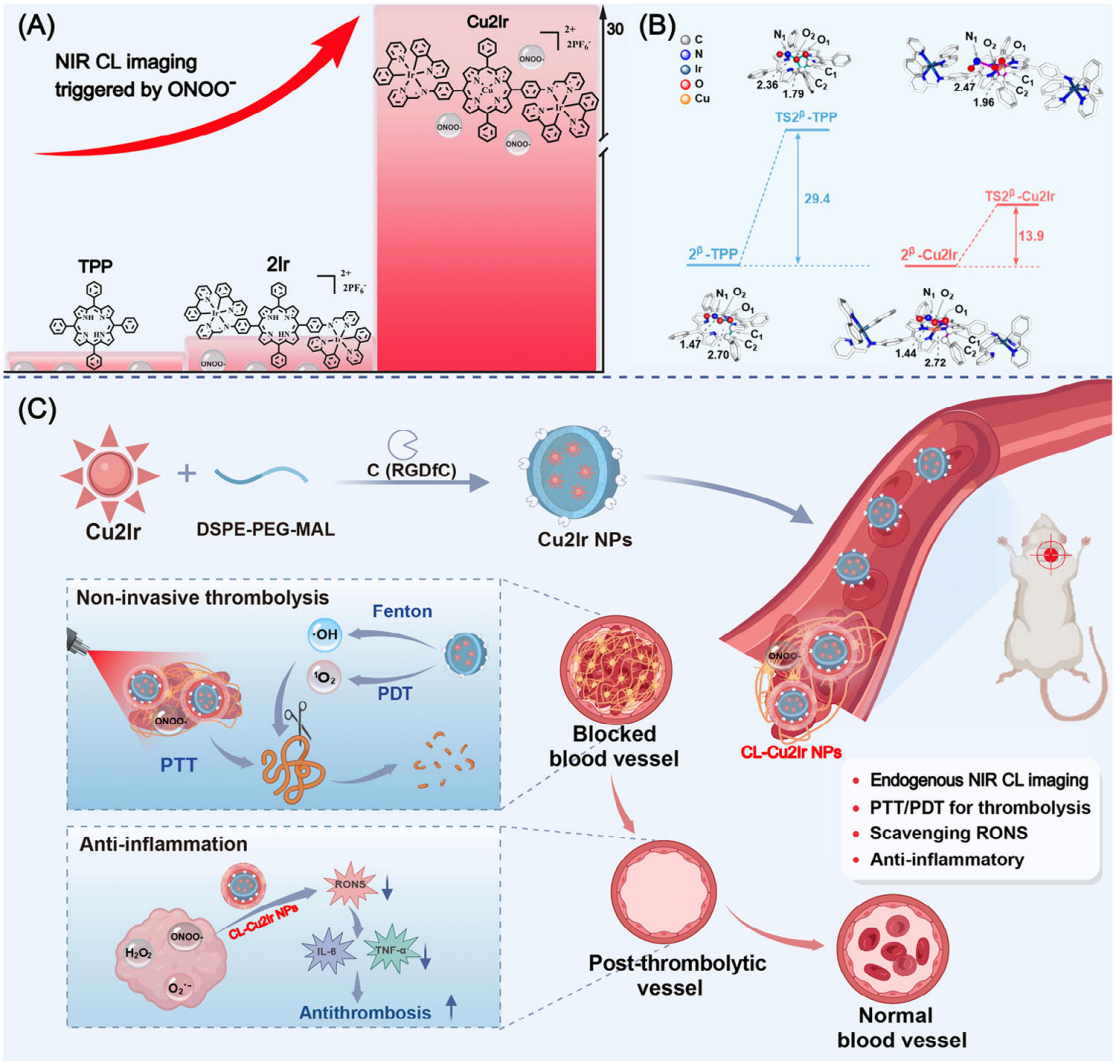
A) Chemical structures of TPP, 2Ir, Cu2Ir and schematic representation of the NIR CL intensity of TPP, 2Ir and Cu2Ir triggered by ONOO^−^. B) Gibbs energy changes (in kcal/mol) of the rate‐determining step in the formation of unstable intermediates of oxidized TPP and Cu2Ir by ONOO^−^ and specific structural information. C) Schematic illustration of thrombotic therapy with Cu2Ir NPs.

Based on the above considerations, this paper reports a heteronuclear copper‐porphyrin complex (Cu2Ir) for the first time which is triggered by endogenous ONOO^−^ to generate strong NIR CL for non‐invasive, safe thrombolytic and anti‐inflammatory therapy. Molecular engineering with the combination of porphyrin and heteronuclear transition metals gives Cu2Ir nanoparticles (NPs) that exhibit more than 30 times NIR CL intensity (at *λ*_max_ 800 nm) than that of TPP NPs (**Scheme** **1**A), with CL lasting for 60 min with deep tissue penetration (12 mm). Based on DFT calculations, the reactive site for the CL of metal‐free porphyrin (TPP) derivatives and the mechanism to enhance the CL properties are revealed for the first time (Scheme 1B). The Cu2Ir NPs realized dual modal PTT/PDT thrombolytic therapy and achieved a thrombolysis rate of 72.3%, efficiently lysing the thrombus without bleeding side effects. Cu2Ir NPs showed remarkable antioxidant abilities, scavenged the abnormal accumulation of RONS and reduced the expression of pro‐inflammatory cytokines, preventing re‐embolism, compared to free UK (Scheme 1C). To the best of our knowledge, this is the first chemiluminescence imaging‐guided, safe thrombolysis, with recurrence prevention and anti‐inflammatory multifunctionality as a one‐for‐all therapeutic strategy.

## 2 Results and Discussion

Cyclometallated Ir(III) complexes have the advantages of long‐lived triplet metal‐ligand charge transfer states, good biocompatibility and desirable effects in PDT and PTT.^[^ ^11^ ^]^ We synthesized the diiridium complex 2Ir with a metal‐free porphyrin core and the derived heteronuclear complex Cu2Ir with the aim of obtaining enhanced chemiluminescent intensities compared to the parent porphyrin derivatives (TPP) (Scheme 1A). The synthetic route to 2Ir and Cu2Ir is shown in Scheme S1 in the Supporting Information. First, TPP‐(NH_2_)_2_ was synthesized by a previously reported method.^[^ ^28^ ^]^ L2 was obtained via a simple Schiff base reaction, and L2 reacted with [Ir(ppy)_2_Cl]_2_ to achieve complex 2Ir. Cu2Ir was then obtained through the reaction of 2Ir and excess Cu(Ac)_2_. The structures of the complexes were verified by ^1^H NMR, ^13^C NMR, mass spectra and FT‐IR spectra (Figures S1–S8, Supporting Information).

To assess the CL properties of TPP, 2Ir, and Cu2Ir, their peroxynitrite oxidation mechanisms were investigated using density functional theory (DFT) calculations (detailed in the Supporting Information). Peroxynitrite was chosen for its high toxicity compared to other RONS, due to its destructive oxidation and nitration reactions with lipids, mitochondria and DNA.^[^ ^30^ ^]^ First, for unsubstituted porphyrin the oxidation pathway of the C═C bond consists of two steps, with the second step identified as the rate‐limiting step, due to the higher **TS2*^β^*** (48.4 kcal mol^−1^) than **TS1*^α^*** (19.8 kcal mol^−1^, Figure S9, Supporting Information).

**Figure 1:**
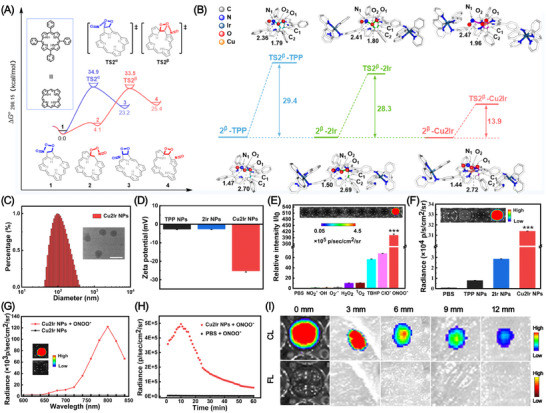
A) Gibbs energy changes (in kcal/mol) of the rate‐determining step in the formation of unstable intermediates by ONOO^−^ oxidizing TPP at different sites (TS = Transition State). B) Gibbs energy changes (in kcal/mol) of the rate‐determining step in the formation of unstable intermediates by ONOO^−^ oxidizing TPP, 2Ir and Cu2Ir and specific structural information. C) DLS analysis and TEM image of Cu2Ir NPs (Scale bar = 200 nm). D) Zeta potential changes of TPP NPs, 2Ir NPs and Cu2Ir NPs in water. E) Selectivity of the preirradiated Cu2Ir NPs toward different RONS (200 µM) treatments. Inset: the CL images acquired by an IVIS imaging system under bioluminescence mode with an open filter. F) CL intensity plot of TPP NPs, 2Ir NPs and Cu2Ir NPs. Inset: the corresponding CL acquired on an IVIS imaging system. G) CL spectra of Cu2Ir NPs in the absence or presence of ONOO^−^ in PBS solution (pH 7.4). Inset: the corresponding CL acquired on an IVIS imaging system. H) Decay of persistent luminescence signal of Cu2Ir NPs (200 µg mL^−1^) and PBS solution (pH 7.4) over time at room temperature after addition of ONOO^−^ (200 µm). I) Tissue penetration depths of the activated CL of preirradiated and ONOO^−^‐added Cu2Ir NPs and the fluorescence (FL) from Cu2Ir NPs excited at 465 nm (in PBS at pH 7.4) with a coverage of chicken breast tissues with different thicknesses. *p* < 0.05 was considered statistically significant. *p < 0.05, **p < 0.01, ***p < 0.001, ****p < 0.0001.

Then, the reactivity of the *α* and *β* C═C bonds in porphyrin was further analyzed, focusing on the second step. As illustrated in **Figure** **1**A, **TS2*^α^*** and **TS2*^β^*** correspond to the transition states for the oxidation of the *α* and *β* C═C bonds, respectively. As expected, **TS2*^β^*** is higher than **TS2*^α^***, indicating that a *β* C═C bond is preferentially oxidized than an *α* C═C bond. To our knowledge this is the first time that the preferred *β* C═C reaction site of the porphyrin ring has been established (see Figure S10, Supporting Information for details).

The mechanisms of the *β* C═C bond oxidation by ONOO^−^ in TPP, 2Ir, and Cu2Ir were explored to predict their variation trend of chemiluminescent properties. Figure 1B and Figure S10 (Supporting Information) shows the trend in energy barriers is Cu2Ir ≪ 2Ir ≪ TPP. Specifically, the energy barrier for Cu2Ir is 14.4 kcal mol^−1^ lower than that of 2Ir and 15.5 kcal mol^−1^ lower than that of TPP, indicating that Cu2Ir is the easiest to react with ONOO^−^ to generate CL. These findings demonstrate that the interaction between the heteronuclear transition metals and porphyrin derivatives is important to significantly enhance the CL.

Then Cu2Ir NPs were formed with the reactive phospholipid DSPE‐PEG‐MAL and functionalized by the C(RGDfC) peptide (Figure S11, Supporting Information). 2Ir NPs and TPP NPs were obtained by the same method.^[^ ^29^ ^]^ C(RGDfC) can bind to activated GPIIb/IIIa receptors on the surface of platelets to ensure thrombus targeting.^[^ ^31^ ^]^ Cu2Ir NPs, 2Ir NPs and TPP NPs were analyzed by transmission electron microscopy (TEM) and dynamic light scattering (DLS). The NPs were circular with particle sizes of 129, 92, and 75 nm, respectively (Figure 1C; Figure S12, Supporting Information). The dimensions measured by DLS are inflated because of a hydrated surface layer on the NPs. Moreover, the diameters of all the NPs exhibit minimal variation after 14‐days immersion in water (Figure S13, Supporting Information). The Cu2Ir NPs have the lowest potential of‐25.2 mV (Figure 1D), uniform round shape and good stability, which are advantageous for their intercellular transport.

NIR CL was observed in vitro as Cu2Ir NPs were oxidized by ONOO^−^. Unlike traditional fluorescence imaging, CL imaging does not require excitation by an external light source, and it has good signal‐to‐noise ratio and superior imaging quality. To better verify the CL properties, a series of RONS were chosen to assess the selectivity: namely NO_2_^−^, *t*‐Bu‐hydroperoxide (TBHP), •OH, O_2_^•−^, ^1^O_2_, H_2_O_2_, ClO^−^, and ONOO^−^. Among the various RONS, Cu2Ir NPs oxidized by ONOO^−^ exhibited the highest CL intensity, which was ≈ 402 times higher than the phosphate‐buffered saline (PBS) group (Figure 1E). As show in Figure 1F and Figure S14 (Supporting Information), Cu2Ir NPs achieved > 30 orders of magnitude CL intensity compared with TPP NPs, which aligns with the DFT calculations that Cu2Ir is the easiest to react with ONOO^−^ to generate CL. In addition, the transition metal (Cu) within the porphyrin ring should also favor Cu2Ir to react with ONOO^−^.^[^ ^27^ ^]^ Although Cu2Ir NPs show the weakest fluorescence intensity, they exhibited the strongest CL intensity compared to 2Ir NPs and TPP NPs. The combination of weak fluorescence and strong CL is consistent with recent reports.^[^ ^32^ ^]^ The CL spectra of Cu2Ir NPs were detected at different emission peak positions and *λ*_max_ is at 800 nm (Figure 1G). The fluorescence spectra of the intermediate products are shown in Figure S15 (Supporting Information), which correspond with the CL of Cu2Ir NPs. Chemiluminescence plots of NPs at different pH are shown in Figure S16 (Supporting Information). From pH 5.0 to 8.5, Cu2Ir NPs exhibited the strongest CL compared to 2Ir NPs and TPP NPs. The CL signals of Cu2Ir NPs showed a linear relationship with the concentration of ONOO^−^, and the correlation coefficient reached 0.9938 (Figure S17, Supporting Information). In addition, the CL intensity was also different after different irradiation times (Figure S18, Supporting Information). As shown in Figure 1H, the intensity for Cu2Ir NPs reached a maximum at 10 min after the addition of ONOO^−^ and then slowly decayed. The CL signal persisted for 60 min and the intensity remained higher than that of the PBS group. The depth of penetration into tissues affects their imaging efficiency when drugs are used for in vivo treatment. We tested the CL penetration depth of Cu2Ir NPs covered with different thickness of chicken breasts. As shown in Figure 1I, CL signals can still be seen when the thickness reached to 12 mm. Mass spectrometric data shows that the reaction products of Cu2IrNPs with ONOO^−^ are aldehydes and ketones; the spectra and the proposed CL reaction mechanism (which is similar to a previous report)^[^ ^24^ ^]^ are shown in Figure S19 (Supporting Information). The remarkably enhanced CL indicated that Cu2Ir NPs show great application potential to monitor the changes in a deep thrombus.

**Figure 2:**
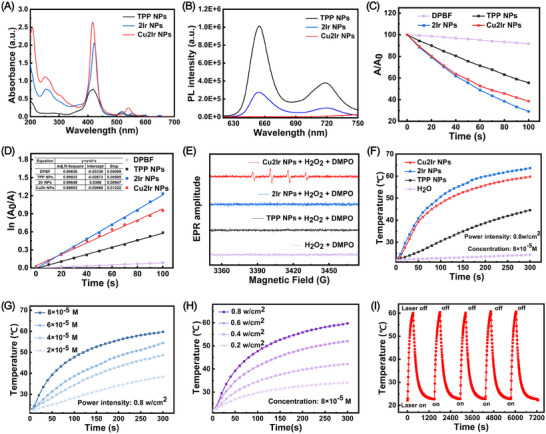
A) UV–vis absorption spectra of TPP NPs, 2Ir NPs and Cu2Ir NPs in water. B) Fluorescence spectra of TPP NPs, 2Ir NPs and Cu2Ir NPs in water. C) Comparison of decay rates of TPP NPs, 2Ir NPs and Cu2Ir NPs under irradiation (635 nm, 0.8 w cm^−2^), A_0_ = DPBF absorption under no irradiation conditions. A = Real‐time uptake of DPBF at different irradiation time. D) ^1^O_2_ generation kinetics over time. E) EPR signals of DMPO (for •OH) with different treatments. F) Photothermal profiles of TPP NPs, 2Ir NPs and Cu2Ir NPs in water under 635 nm laser irradiation. G) Concentration and H) Laser power density dependence of the photothermal effects of Cu2Ir NPs under 635 nm laser irradiation. I) Temperature variations of the Cu2Ir NPs (8 × 10^−5^ m) under irradiation at a power density of 0.8 w cm^−2^ for five light on/off cycles.

The UV‐vis absorption and photoluminescence (PL) spectra of TPP NPs, 2Ir NPs and Cu2Ir NPs were recorded in water (**Figure** **2**A,B). The extended absorption band in the 500–700 nm region is advantageous for matching long‐wavelength excitation to enhance tissue penetration. It is noteworthy that the PL intensity of 2Ir NPs is significantly less than that of the TPP NPs,^[^ ^11^ ^]^ while the incorporation of the paramagnetic Cu in the porphyrin ring results in barely detectable fluorescence. We evaluated the PDT performance of TPP NPs, 2Ir NPs and Cu2Ir NPs. Utilising 1,3‐diphenylisobenzofuran (DPBF) as an indicator, the absorbance at 415 nm of DPBF under 635 nm laser irradiation was monitored by UV‐Vis absorption spectra (Figures S20–S23, Supporting Information). The absorbance was significantly reduced in the presence of Cu2Ir NPs, which demonstrated their good ^1^O_2_‐generating ability. From the decay curves in Figure 2C and the first‐order kinetics of ^1^O_2_ production in Figure 2D, the photocatalytic performance of the 2Ir NPs is stronger than that of the TPP NPs, indicating that the introduction of the two Ir complexes is beneficial for the generation of ^1^O_2_. However, Cu2Ir NPs show slightly weaker ^1^O_2_ generation ability than 2Ir NPs which is probably due to the slightly decreased absorption properties of 2Ir NPs (Figure 2A). Notably, electron spin resonance (ESR) spectra of Cu2Ir NPs in the presence of H_2_O_2_ and 5,5‐dimethyl‐1‐pyrroline‐*N*‐oxide (DMPO) as probe show the production of •OH radicals, not observed with TPP NPs or 2Ir NPs (Figure 2E). This is attributed to a Fenton‐like reaction (chemodynamic therapy, CDT) between copper in the Cu2Ir NPs and H_2_O_2_. Therefore, the combination of heteronuclear transition metal complexes and porphyrin derivatives gives excellent ROS‐generating ability, which predicts Cu2Ir NPs’ potential for exceptional PDT thrombolysis.

The photothermal properties of the NPs were also investigated in water. Upon 635 nm laser irradiation, TPP NPs induced a prominent temperature increase from room temperature to 44.5 °C in 300 s, whereas the temperature of pure water barely changed under the same conditions. As expected, Cu2Ir NPs and 2Ir NPs showed a strong photothermal effect, reaching 59.7 °C and 63.6 °C, respectively, confirming the benefits of the metal centres (Figure 2F). The photothermal conversion efficiency of Cu2Ir NPs is 45.61% (Figure S24, Supporting Information) and the associated calculations are reported in the Supporting Information. In addition, temperature changes in solution at different irradiation times were also recorded with an infrared camera (Figure S25, Supporting Information). Concentration and laser intensity‐dependent temperature changes are shown in Figure 2G,H. These data clearly indicate the prominent photothermal effects of Cu2Ir NPs. Figure 2I shows the temperature of Cu2Ir NPs solution (8 × 10^−5^ m) during five on/off switching cycles of the laser irradiation (0.8 w cm^−2^) demonstrating high photothermal stability. It is concluded that Cu2Ir NPs have both excellent PTT and PDT performance, which is highly favorable for non‐invasive thrombolysis.

**Figure 3:**
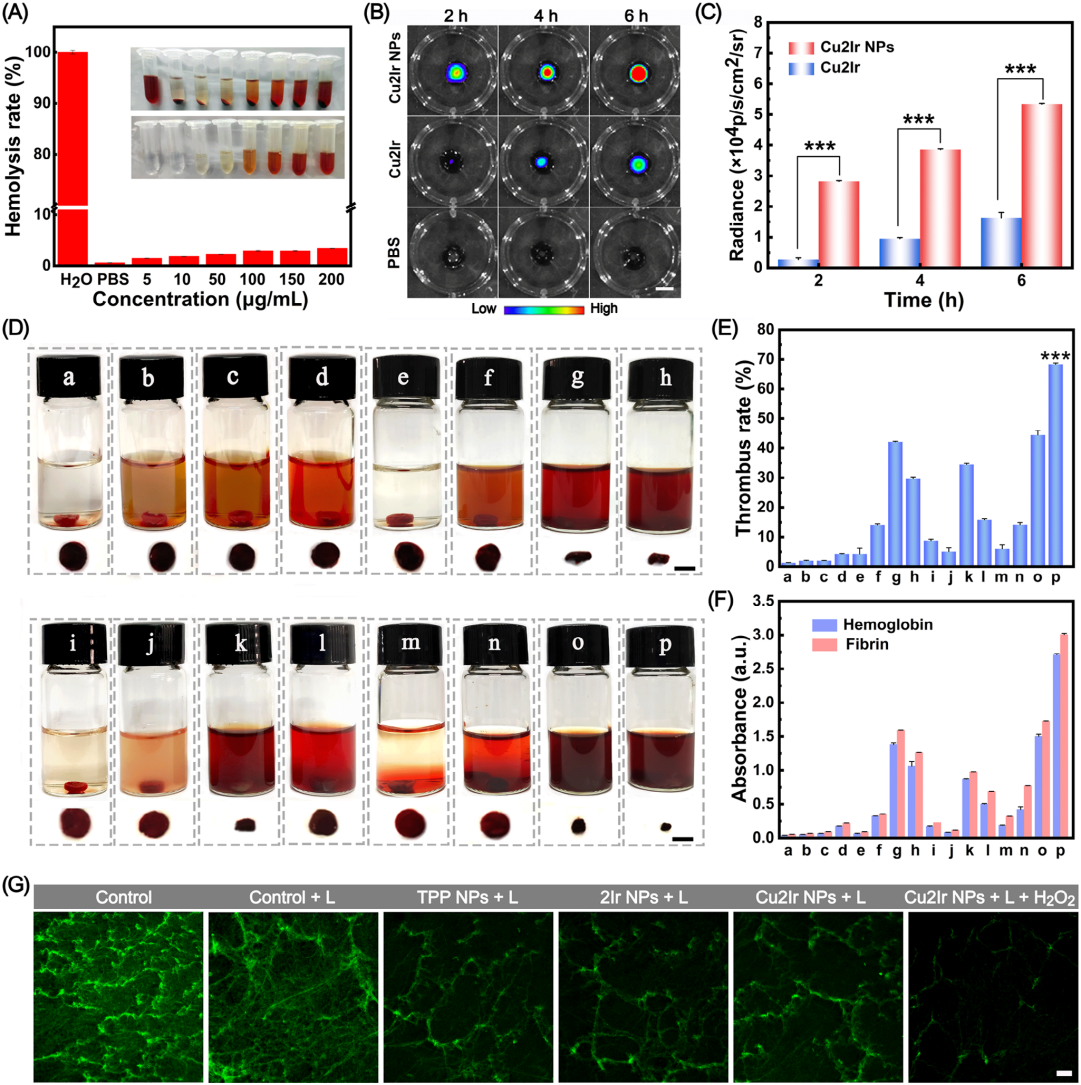
A) The hemolysis rate of red blood cells treated with water and different concentrations of Cu2Ir NPs. Inset shows samples in the presence or absence of the red blood cells solution upon different treatments (from left to right: water, PBS, 5, 10, 50, 100, 150, and 200 µg mL^‒1^ of Cu2Ir NPs;NPs not co‐incubated with erythrocytes are illustrated below. Data are presented as mean ± SD (n = 3 independent experiments). B) The CL images of artificial blood clots after incubation with PBS, Cu2Ir or Cu2Ir NPs. Scale bar = 5 mm. C) Quantitative analysis of the relative CL intensity of the artificial blood clots in (B). D) Photographs of the blood clot solution after different treatments (a: PBS, b: TPP NPs, c: 2Ir NPs,d: Cu2Ir NPs, e: PBS + L (light), f: TPP NPs + L, g: 2Ir NPs + L, h: Cu2Ir NPs + L, i: PBS + Vc (vitamin C) + L, j: free UK (urokinase), k: Cu2Ir NPs + Vc + L, l: Cu2Ir NPs + 0 °C + L, m: PBS + H_2_O_2_ +L, n: TPP NPs + H_2_O_2_ + L, o: 2Ir NPs + H_2_O_2_ + L and p: Cu2Ir NPs + H_2_O_2_ + L). The residual blood clots were taken out and shown below. Scale bars = 5 mm. E) The quantified clot‐dissolution efficiency of different treatment groups (corresponding to Figure 3D) in vitro. F) The absorbance at 450  and 540 nm and in different treatment groups (corresponding to Figure 3D) after 50 min of treatment. G) CLSM images of fibrin clot after incubating PBS with or without irradiation, and TPP NPs, 2Ir NPs, Cu2Ir NPs with irradiation, Cu2Ir NPs with irradiation and H_2_O_2_. Scale bar = 100 µm. *p* < 0.05 was considered statistically significant. *p < 0.05, **p < 0.01, ***p < 0.001, ****p < 0.0001.

Encouraged by the photophysical properties of Cu2Ir NPs, we explored their thrombolytic capacity in vitro. Red blood cells (RBCs) are the most abundant cells in blood. The blood compatibility of NPs was assessed by calculating the percentage of hemolysis through the capacity of the Cu2Ir NPs to rupture the RBCs. RBCs in PBS and in water were used as negative and positive controls, respectively. **Figure** **3**A shows that the hemolysis rate remained at <5% even when the Cu2Ir NPs concentration reached 200 µg mL^−1^, which proved that the Cu2Ir NPs did not destroy the RBCs and they possess outstanding blood compatibility. To start the thrombolytic test, the targeting specificity of Cu2Ir NPs to thrombus was examined with in vitro blood clots. The clots were prepared by mixing fresh mouse blood with thrombin, and the chemiluminescent properties of Cu2Ir NPs enable imaging of the blood clots. After the incubation of the clots with Cu2Ir NPs, non‐targeted Cu2Ir, and PBS for 2, 4 or 6 h, the clots were visualized with an in vivo imaging system (IVIS). Indeed, the clots incubated with Cu2Ir NPs exhibited a remarkably stronger CL signal than those of the Cu2Ir and PBS groups at each time point (Figure 3B). The CL intensities of Cu2Ir NPs and Cu2Ir are quantified in Figure 3C. The Cu2Ir NPs mediated by C(RGDfC) peptide show clearly improved thrombus targeting properties.

The thrombolytic effects of various formulations were then investigated by subjecting the blood clots to different treatments, i.e., a: PBS, b: TPP NPs, c: 2Ir NPs, d: Cu2Ir NPs, e: PBS + L (light), f: TPP NPs + L, g: 2Ir NPs + L, h: Cu2Ir NPs + L, i: PBS + Vc (vitamin C) + L, j: free UK (urokinase), k: Cu2Ir NPs + Vc + L, l: Cu2Ir NPs + 0 °C + L, m: PBS + H_2_O_2_ + L, n: TPP NPs + H_2_O_2_ + L, o: 2Ir NPs + H_2_O_2_ + L and p: Cu2Ir NPs + H_2_O_2_ + L (Figure 3D). In all the laser‐treated groups, the thrombolysis rate of group (k) with only PTT thrombolysis was 19% higher than that of group (l) with only PDT thrombolysis, which proved that PTT dominated the process. Compared with the Cu2Ir NPs group (h), the thrombolysis rate of 2Ir NPs group (g) under laser irradiation was slightly higher, which may be due to the higher local heat generated by the 2Ir NPs under light irradiation in photothermal testing. After H_2_O_2_ was added (which is in excess in a thrombus microenvironment) to co‐incubate with the NPs, the thrombolytic effect was greatly enhanced in the Cu2Ir NPs group (p) compared with group (o) and the thrombolytic rate reached to ∼70% (Figure 3E). Also, the amount of hemoglobin and fibrin released into the supernatant during clot lysis presented a similar trend with lysis efficiency (Figure 3F). These results all predict that Cu2Ir NPs will achieve marked thrombolysis in vivo. Further, to explore the disruptive effect of RONS on the fibronectin skeleton, the amount of collapsed skeleton and fragments were distinguished after incubating fibrin with NPs and light irradiation (Figure 3G). Especially for the Cu2Ir NPs + L + H_2_O_2_ group, the fragments of fibrin were almost completely destroyed. This may be attributed to the •OH produced by Cu2Ir NPs reacting with H_2_O_2_ to further disrupt the fibrin backbone, thereby effectively improving the thrombolytic efficacy. These in vitro data indicate that Cu2Ir NPs have good biocompatibility and thrombus targeting, as well as excellent PTT/PDT performance, which predicts their excellent thrombolytic efficacy in vivo.

**Figure 4:**
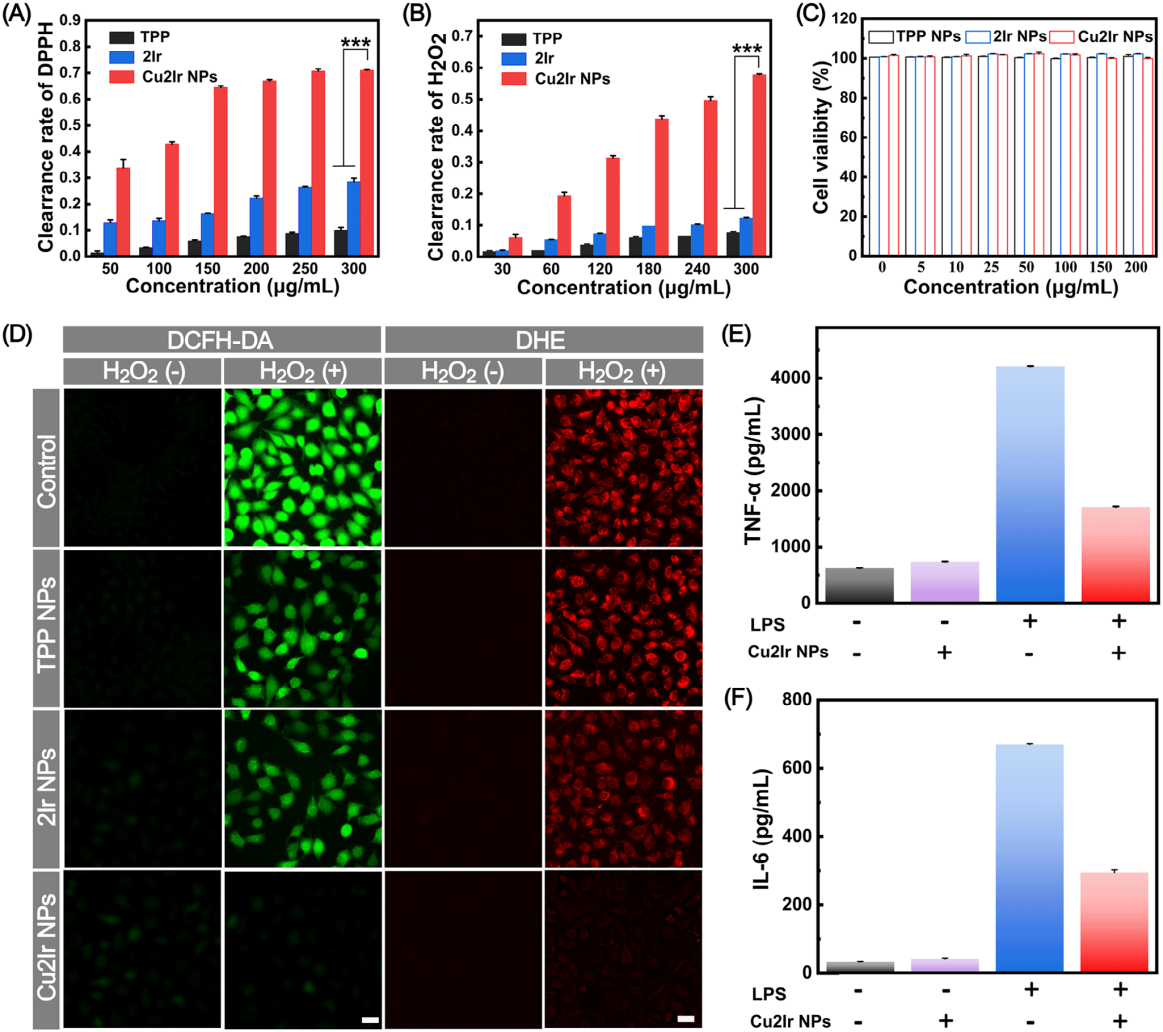
A) DPPH scavenging activity of TPP, 2Ir and Cu2Ir. B) H_2_O_2_ scavenging ability of TPP, 2Ir and Cu2Ir. C) Cell viability was measured by MTT assay after HUVEC cells were treated with different concentrations of NPs for 24 h. D) NPs scavenged RONS and O_2_^•−^ activity induced by H_2_O_2_‐treated HUVEC cells in vitro. Scale bars = 50 µm. Concentrations of E) TNF‐α and F) IL‐6 were determined by an enzyme‐linked immunosorbent assay (ELISA). *p* < 0.05 was considered statistically significant. *p < 0.05, **p < 0.01, ***p < 0.001, ****p < 0.0001.

Next, we used 2,2‐diphenyl‐1‐(2,4,6‐trinitrophenyl)hydrazyl (DPPH) and H_2_O_2_ to evaluate the antioxidant capacity of TPP, 2Ir and Cu2Ir. The strongest scavenging ability was observed for Cu2Ir in a dose‐dependent manner without laser irradiation; the scavenging rate is 71.0% for DPPH and 57.8% for H_2_O_2_ at the concentration of 300 µg mL^−1^ (**Figure** **4**A,B), while TPP and 2Ir show negligible RONS scavenging ability. These results suggest that Cu2Ir possesses the most versatile and the best antioxidant properties among the series. The construction of heteronuclear transition metal complexes endows this property to porphyrin derivatives.

As shown in Figure 4C, the cytotoxicity of NPs in human umbilical vein endothelial (HUVEC) cells was measured by a 3‐(4,5‐dimethylthiazol‐2‐yl)‐2,5‐diphenyltetrazolium bromide (MTT) assay. As expected, the cells maintained a high survival rate (>95%) even at a concentration of NPs as high as 200 µg mL^−1^ of the NPs, revealing the excellent biosafety of the NPs. Then the ability of NPs to scavenge RONS in HUVEC cells was investigated. After treatment with 400 mΜ H_2_O_2_, the level of RONS (green fluorescence intensity) significantly increased in HUVEC cells as detected by 2′,7′‐dichlorodihydrofluorescein diacetate (DCFH‐DA) assay. In contrast, RONS levels were markedly reduced in the Cu2Ir NPs group compared with the 2Ir NPs and TPP NPs groups. Meanwhile, the ability of NPs for scavenging superoxide anion (O_2_^•−^) was probed by dihydroethidium (DHE) assay; the reduced intensity of red fluorescence in the cells demonstrated that the NPs could scavenge O_2_^•−^. As shown in Figure 4D, the Cu2Ir NPs showed the lowest intensity of red fluorescence, which implies the strongest ability to scavenge O_2_^•−^ of the NPs. The quantitative analysis of the fluorescence intensity is shown in Figure S26 (Supporting Information). Nevertheless, negligible fluorescence intensity variation was observed between TPP NPs and 2Ir NPs. Overall, these results suggest that Cu2Ir NPs are effective RONS scavengers.

Inspired by the above results, we examined the anti‐inflammatory effect of Cu2Ir NPs. First, 1 µg mL^−1^ of lipopolysaccharides (LPS) was used to induce the activation of RAW 264.7 cells. Cu2Ir NPs exhibited a superior anti‐inflammatory activity as both tumour necrosis factor–α (TNF‐α) and interleukin‐6 (IL‐6) showed significantly decreased expression levels (Figure 4E,F). The significant RONS‐scavenging and anti‐inflammatory capacity of Cu2Ir NPs should provide a positive prognosis for post‐thrombolytic blood vessels.

**Figure 5:**
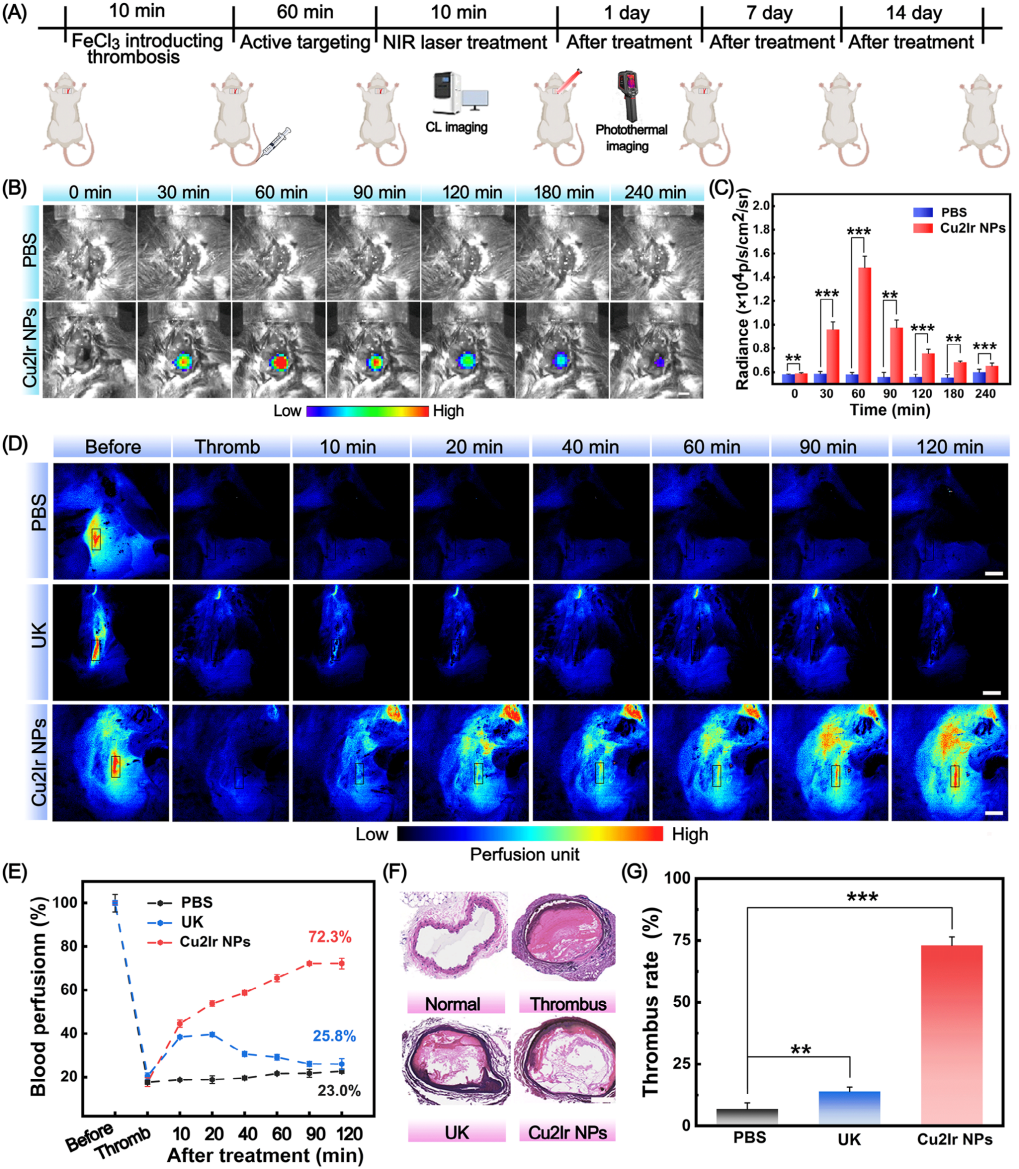
A) Carotid artery thrombosis modeling and thrombolytic treatment protocols. B) Representative in vivo CL images of the thrombotic artery at different timepoints post‐injection of PBS and Cu2Ir NPs. Scale bar = 5 mm. C) Quantification of the relative CL intensity of mouse neck thrombi in (B). D) During Laser Speckle Blood Flow Monitoring System (LSBFMS) imaging, the right carotid thrombotic artery of each mouse (indicated with black lines) was focused, and its blood perfusion was quantified. Scale bars = 5 mm. E) Representative LSBFMS images and the corresponding relative blood perfusion of the mouse carotid artery after FeCl_3_ induction and different therapeutic treatments, including PBS, free UK, Cu2Ir NPs. Data are presented as mean ± SD (n = 3 mice). F) Hematoxylin and eosin (H&E) staining of vessels after different treatments. Scale bar = 200 µm. G) Quantitative statistics of thrombus area for different groups. *p* < 0.05 was considered statistically significant. *p < 0.05, **p < 0.01, ***p < 0.001, ****p < 0.0001.

The potential of Cu2Ir NPs to treat obstructive thrombosis was investigated in vivo according to the experimental scheme in **Figure** **5**A. Imaging tests assessed the target properties of Cu2Ir NPs in thrombi. A group of mice with FeCl_3_‐induced thrombus was injected with Cu2Ir NPs (5 mg kg^−1^), while the control group received PBS in the same volume. As shown in Figure 5B,C, the CL signal in the thrombus was significantly enhanced after injection of Cu2Ir NPs within 60 min. These findings suggest that the CL generated by Cu2Ir NPs can accurately pinpoint the thrombus. Upon laser irradiation, the temperature at the thrombus site increased, while there were only negligible temperature changes in the adjacent tissue. These changes were also recorded using an infrared camera (Figure S27, Supporting Information). Superior CL imaging and photothermal imaging ensured that the variation of the thrombus was easily tracked. Then, we evaluated the thrombolytic ability of Cu2Ir NPs in a mouse model with carotid artery thrombosis. The blood vessels were differently treated with PBS, free UK and Cu2Ir NPs, which were separately administrated into the mice via the tail vein. Laser Speckle Blood Flow Monitoring System (LSBFMS) images clearly showed the cessation of blood perfusion at the time of thrombus formation and changes in blood flow during the treatment (Figure 5D; Figure S28, Supporting Information). Free UK (0.04 mg kg^−1^) was able to dissolve the blood clot and restored the blood flow to some extent, but the blood flow decreased again after 40 min, which was probably due to the short lifetime of macromolecular protein drugs (like UK) in blood. Mice injected with Cu2Ir NPs and PBS in the tail vein were treated with 635 nm (0.8 w cm^−2^) laser irradiation. The excellent PTT property of Cu2Ir NPs produced site‐specific high temperatures at the thrombus site, thereby effectively dissolving the blood clot. At the same time, the PDT property of Cu2Ir NPs could destroy the fibrin skeleton in the blood clot. Consequently, the blood flow in the mice was recovered and gradually stabilized, and no bleeding side effects after reperfusion were observed for Cu2Ir NPs. The final thrombolysis rate was 72.3% with no re‐embolism (Figure 5E). These data indicate the great capacity of Cu2Ir NPs to efficiently reverse thromboembolism in the right carotid thrombotic artery of mice (Figure S29, Supporting Information).

Histological examination of carotid tissues was performed by hematoxylin and eosin (H&E) staining (Figure 5F). In contrast to embolized vessels, the vessels treated with Cu2Ir NPs exhibited lower levels of erythrocytes and platelets, a loser fibrin scaffold and less inflammatory cell infiltration, which is the closest staining to a normal blood vessel compared to the free UK group. Quantitative statistics of the thrombus area in H&E staining of the dissected vessels demonstrated that Cu2Ir NPs achieved a larger dissolution of thrombolytic area than that of free UK (Figure 5G). This illustrates that Cu2Ir NPs present remarkable antithrombotic activity in vivo and provide an effective molecularly engineered protocol for preventing re‐embolism, giving a favorable prognosis.

Further study of the safety of Cu2Ir NPs in mice in vivo involved tail vein injection of PBS, UK, and Cu2Ir NPs. There was no significant change in body weight observed within 14 days (Figure S30, Supporting Information). The survival rate of mice within 14 days is shown in Figure S31 (Supporting Information). Major organs of the mice, including the brain, heart, liver, spleen, lung, and kidney, were collected for post‐mortem examination (Figure S32, Supporting Information). The H&E tissue staining graph (Figure S33, Supporting Information) shows that none of the organs has obvious pathological changes or inflammatory reactions after treatment with Cu2Ir NPs. At the same time, comprehensive blood tests were performed on the mice, and the results showed that the hematological indexes of those treated with Cu2Ir NPs were within the normal range (Figure S34, Supporting Information). Taken together, these results revealed the excellent biocompatibility of Cu2Ir NPs in vivo, which is very important for safe thrombolysis.

## 3 Conclusion

In summary, our research presents the heteronuclear transition metal complex Cu2Ir NPs with the following advantages: 1) endogenous NIR chemiluminescent imaging; 2) non‐invasive thrombolysis; 3) RONS scavenging and anti‐inflammation. For the first time, by DFT calculations, it is revealed that the CL reaction site between TPP and ONOO^−^ occurs at the *β* C═C bonds and that a heteronuclear transition metal complex greatly enhances TPP's CL properties. Cu2Ir NPs achieved nearly 30 times enhancement of CL intensity compared with TPP NPs. Additionally, a tissue penetration depth of 12 mm for the NIR CL signal was realized, with a sustained CL lasting 60 min, which enables real‐time, accurate detection of thrombotic lesions with an endogenous ONOO^−^ response. The Cu2Ir NPs were activated to achieve dual‐mode PTT/PDT thrombolysis under 635 nm laser irradiation. This treatment effectively dissolved the thrombus without the risk of bleeding and successfully prevented secondary embolism. After light irradiation, the Cu2Ir NPs scavenged the harmful RONS, reduced inflammatory factors and restored the local microenvironment of the vessels. Remarkably, the autonomous antioxidant capacity of Cu2Ir NPs effectively prevented re‐embolization and provided a favorable prognosis for thrombolysis of the vessels. This work demonstrates a viable strategy that combines diagnostic, safe thrombolytic, antioxidant, and anti‐inflammatory properties in a single molecular complex to realize a multi‐stage thrombolytic strategy. Overall, this work represents a landmark paradigm in one‐for‐all “smart” nanomedicines for future clinical trials in thrombosis therapy.

## Conflict of Interest

The authors declare no conflict of interest.

## Supporting information



Supporting Information

## Data Availability

The data that support the findings of this study are available in the supplementary material of this article.
