# Supplementary material for: Endogenous Near‐Infrared Chemiluminescence: Imaging‐Guided Non‐Invasive Thrombolysis and Anti‐Inflammation Based on a Heteronuclear Transition Metal Complex
Source: Adv Sci (Weinh). 2025 Mar 26;12(19):2501257. doi: 10.1002/advs.202501257 (PMC12097061; doi:10.1002/advs.202501257)
Supplement: Supplementary file 1 — Supporting Information [file ADVS-12-2501257-s001.pdf]

ADVANCED SCIENCE
Open Access

Supporting Information

for *Adv. Sci.*, DOI 10.1002/advs.202501257

Endogenous Near-Infrared Chemiluminescence: Imaging-Guided Non-Invasive Thrombolysis and Anti-Inflammation Based on a Heteronuclear Transition Metal Complex

Ziwei Wang, Bo Zhu, Wenxin Nie, Liping Zhang, Nan Xiao, Qiaohua Zhang, Zihan Wu, Chunguang Shi, Weijin Zhu, Qianwen Liu, Dongxia Zhu*, Martin R. Bryce*, Lijie Ren* and Ben Zhong Tang**

Supporting Information
©Wiley-VCH 2024
69451 Weinheim, Germany

Endogenous Near-Infrared Chemiluminescence: Imaging-Guided Non-Invasive Thrombolysis and Anti-Inflammation Based on a Heteronuclear Transition Metal Complex

Ziwei Wang+[a], Bo Zhu+[a], Wenxin Nie+ [a], Liping Zhang*[b], Nan Xiao[b], Qiaohua Zhang[a], Zihan Wu[a], Chunguang Shi[a], Weijin Zhu[a], Qianwen Liu[a], Dongxia Zhu*[a], Martin R. Bryce*[c], Lijie Ren* [b] and Ben Zhong Tang* [d,e]

Abstract: Conventional therapy to treat thrombi (blood clots) has significant limitations: i) inflammation; ii) bleeding side effects; iii) re-embolisation, and iv) in situ thrombi that are not visible. Here we report that Cu2Ir nanoparticles (NPs) with a Cu-coordinated tetraphenylporphyrin (TPP) core and cyclometalated $Ir(C^\wedge N)_2(N^\wedge N)$ substituents integrate long-lived near-infrared (NIR) chemiluminescence (CL) imaging, photothermal therapy (PTT) and photodynamic therapy (PDT) for thrombolysis, with antioxidant and anti-inflammatory properties. Based on density functional theory calculations the chemiluminescent reaction site between TPP and peroxynitrite ($ONOO^-$) is confirmed for the first time. The presence of the transition metal significantly improves the chemiluminescent properties of TPP. Upon specific activation by $ONOO^-$, Cu2Ir NPs exhibited more than 30-fold NIR CL intensity than TPP NPs, and the luminescence lasted for 60 min allowing for precise and long-lasting dynamic tracking of thrombi. Cu2Ir NPs achieved non-invasive safe thrombolytic therapy triggered by NIR irradiation at the signaling site. 72.3% blood reperfusion was obtained for nearly complete restoration of blood flow, and re-embolism was prevented in a mouse carotid artery model. Furthermore, Cu2Ir NPs scavenged excess reactive oxygen/nitrogen species (RONS) and reduced inflammatory factors. Cu2Ir NPs hold promise as a single molecule strategy for diagnosing and treating diseases associated with thrombosis.

SUPPORTING INFORMATION

Table of Contents

Table of Contents	Page
**Experimental Procedures**	S2
**Computational Methods**	S2
**Synthesis and Characterization**	S4
**Scheme S1.** The synthetic routes.	S4
**Figure S1-S7.** NMR and HRMS spectra of intermediates and products.	S8
**Figure S8.** FT-IR spectra of 2Ir and Cu2Ir.	S9
**Figure S9-S10.** The mechanistic diagram of $ONOO^-$ reacting at different sites of TPP.	S9
**Figure S11.** ^1H NMR spectra of DSPE-PEG-MAL and DSPE-PEG-C(RGDfC).	S9
**Figure S12.** Size and TEM image of TPP NPs and 2Ir NPs.	S11
**Figure S13.** Stability of size distribution changes over 14 days for TPP NPs, 2Ir NPs and Cu2Ir NPs.	S11
**Figures S14-S15**. The persistent luminescence intensities under laser irradiation.	S11
**Figure S16-S19.** Photodynamic characterization of NPs.	S12
**Figure S20-S21.** Photothermal characterization of NPs.	S13
**Figure S22.** Representative LSBFMS images of mice in vivo.	S14
**Figure S23.** Pictures of in vivo thrombolysis in mice.	S14
**Figure S24.** Body weight variations of thrombolytic mice after various treatments.	S15
**Figure S25.** Images of excised tissue from major organs of mice.	S15
**Figure S26-S27.** The H&E and routine blood test data of the mice with different treatments.	S15
**References**	S16

Experimental Procedures

General Materials and Methods.

All the chemicals were obtained from Sigma-Aldrich unless otherwise specified. The solvents for chemical reactions were distilled before use. Materials for organic synthesis were purchased from Energy Chemical Company. 1,2-Distearoyl-sn-glycero-3-phosphoethanolamine-*N*-[maleimide(poly(ethyleneglycol))-2000] (DSPE-PEG-MAL) were purchased from Laysan Bio, Inc. (Arab, AL). C(RGDfC) was supplied by Hangzhou Special Peptide Biotechnology Co. 1,3-Diphenylisobenzofuran (DPBF) was purchased from Energy Chemical Company. Dulbecco's Modified Eagle's Medium (DMEM) was purchased from Solarbio Life Science Company. Urokinase (UK, 1.2 × 105 IU/mg) was obtained from Aladdin Biochemical Technology Co, Ltd. (Shanghai, China) Fetal bovine serum (FBS) was purchased from Sigma-Aldrich. 3-(4,5-dimethyl-2-thiazolyl)-2,5-diphenyl-2*H*-tetrazolium bromide (MTT) and 2'-7'-dichlorofluorescence diacetate (DCFH-DA) were purchased from Shanghai Beyotime Biotechnology Co.Ltd. Enzyme-linked immunosorbent assay (ELISA) kit was provided by Biolegend.

Reactions were monitored with analytical thin-layer chromatography (TLC) on silica. ^{1}H NMR spectra were recorded at 25 °C on a Varian 600 MHz spectrometer. UV-vis absorption spectra were recorded on a Shimadzu UV-3100 spectrophotometer. The photoluminescence spectra were recorded on an Edinburgh FLS920 spectrofluorimeter under air at room temperature. Transmission electron microscopy (TEM) images were taken by a TECNAI F20 microscope. Diameter and diameter distribution of the nanoparticles were determined by a Malvern Zetasizer Nano instrument for dynamic light scattering (DLS). Confocal laser scanning microscopy (CLSM) images were taken using a LSM 800 Zeiss, Germany. Chemiluminescence and fluorescence were acquired by an in vivo imaging system (Xenogen IVIS Lumina II system).

Statistical Analysis. Data are denoted as the mean ± standard deviation (SD). The significance between experimental and control groups was determined by unpaired 2-tailed t-test using the GraphPad Prism 7. A value of $p < 0.05$ was considered statistically significant. *$p < 0.05$, **$p < 0.01$, ***$p < 0.001$, ****$p < 0.0001$.

Computational Methods

The geometry optimizations were carried out using the B3LYP functional. The SMD was used to evaluate solvent effects of water (H_2O). The LANL2DZ basis sets were employed for Cu and Ir atoms with corresponding effective core potentials (ECPs) representing their core electrons and the 6-31G(d) basis sets were used for other main-group elements, which is named BS-I hereinafter. In order to ensure whether it is in an equilibrium structure or in a transition state, and obtain the thermodynamic corrections to the Gibbs energy, the vibrational frequency of each stationary structure was calculated at 298.15 K and 1 atm at the same computational level. An intrinsic reaction coordinate (IRC) was calculated to make sure that a transition state connects a corresponding reactant and product. Single-point calculations were performed to refine potential energies using the same functional with a better basis set (BS-II). In BS-II, the SDD basis sets were employed for Cu and Ir atoms with the corresponding effective core potentials (ECPs) representing their core electrons and the 6-311G(d,p) basis sets were used for others.

Preparation of NPs.

The nanoparticles were prepared by a modified nanoprecipitation method. The synthesis process was carried out at 25 ^oC. First, Cu2Ir (1 mg) and DSPE-PEG-MAL (2 mg) were dispersed in 1 mL of THF. After the compounds were completely dissolved, the above stock solution was added dropwise to 10 mL of purified water under vigorous stirring. Stirring overnight in a fume hood evaporated the organic solvent. Then, C(RGDfC) was dispersed in 1 mL of water and the peptide was loaded by thiol-Michael addition reaction for 16 h at room temperature. The residual C(RGDfC) peptide was then removed using a dialysis bag. The obtained Cu2Ir NPs were stored in a refrigerator at 4 °C for subsequent experiments. 2Ir NPs and TPP NPs were prepared in the same way.

Preparation of Reactive Oxygen and Nitrogen Species (RONS) Solutions.

The RONS solutions containing H_2O_2, TBHP and ClO^-, respectively, were purchased and diluted to the experimental concentration (200 μM) by 1×phosphate buffered saline (PBS) (pH 7.4).[1] In brief, •OH was produced by addition of ferrous chloride (0.1 M, 1 mL) into H_2O_2 solution (1.0 M, 1 mL) through a Fenton reaction. Accordingly, the concentration of •OH is the same as that of Fe^{2+} (50 mM). $O_2^{\bullet-}$ was generated from KO_2 (35.5 mg), which was directly added into dimethyl sulfoxide (10 mL) at a final concentration of 50 mM. $ONOO^-$ was prepared by addition of sodium hydroxide (1.5 M) into mixtures of sodium nitrite (0.6 M), hydrogen peroxide (0.7 M) and hydrochloric acid (0.6 M) at 0 °C, followed by purification through a short column of manganese dioxide to remove excess hydrogen peroxide. The concentration of $ONOO^-$ was determined by measurement of the absorption at 302 nm. $c[ONOO^-] = Abs_{302\ nm}/1.67$ (mM). $^1O_2$ was produced by addition of ClO^- solution (100 mM, 1 mL) into H_2O_2 solution (200 mM, 1 mL). According to this reaction, the concentration of $^1O_2$ is the same as that of ClO^- (50 mM). NO_2^- was generated from $NaNO_2$ (35 mg), which was directly added into deionized water (10 mL) at a final concentration of 50 mM.

Chemiluminescence and Fluorescence Imaging In Vitro.

For in vitro chemiluminescence imaging, including determinations of RONS selectivity, chemiluminescence spectra, and ONOO− activated sensitivity, etc., the concentration of Cu2Ir NPs was 100 μg/mL. The chemiluminescence imaging was carried out with an Xenogen IVIS Lumina II system in a bioluminescent mode (exposure time for 60 s) post RONS (200 μM) addition, unless otherwise specified. Tests were every 20 nm/step by the different filters. The environmental temperature for in vitro afterglow imaging was kept at 37 °C. The fluorescence spectra of Cu2Ir NPs were measured by the same IVIS instrument in a fluorescent mode with excitation at 465 ± 10 nm (exposure time for 1 s). The fluorescence and chemiluminescence images were analyzed by region of interest (ROI) analysis using Living Image 4.2 Software.

Photothermal Conversion Efficiency of the Cu2Ir NPs.

Photothermal conversion efficiency of the Cu2Ir NPs was calculated according to a reported method.[2] First, the Cu2Ir NPs (8×10^{-5} M) in solution were irradiated with 635 nm laser (0.8 W cm^{-2}) for 300 s and then the laser was turned off. After about 1400 s, the solution was cooled to room temperature. The photothermal conversion efficiency (η) was calculated according to the following equation

$$\eta = [hA(T_{Max}-T_{surr})-Q_{dis}]/I(1-10^{-A}) \quad (1)$$

where h and A respectively represent the heat transfer coefficient and the surface area of the container, T_{Max} and T_{sur}r represent the maximum temperature and the room temperature of the environment, Q_{dis} represents the heat dissipation of the solvent (water), I is the laser power employed (0.8 W cm^{-2}), and A is the absorbance of Cu2Ir NPs at 635 nm. The value of hA is calculated from the following equation

$$\tau_s = m_D c_D / hA \quad (2)$$

where τ_s is the time constant for heat transfer of the system, which was determined to be τ_s = 279.42 from Figure S24; m_D and c_D are the mass and heat capacity, respectively, of the deionized water used to disperse the NPs. Thus, the hA was determined to be 0.0045 W. Q_{dis} represents the heat dissipation from the laser absorbed by the water, so Qdis was calculated according to the following equation

$$Q_{dis} = m_D c_D\ (T_{Max\ water} - T_{surr}) /\ \tau_{s\ water} \quad (3)$$

where $T_{Max\ water}$ is the highest temperature of water and $\tau_{s\ water}$ is 168.2; thus, Q_{dis} was calculated to be 0.545 W. According to the obtained data and Equation (1), the photothermal conversion efficiency of the Cu2Ir NPs was determined to be 45.61%.

In Vitro Photothermal Measurements.
To investigate the photothermal performance of the NPs, different NPs aqueous dispersions (1 mL) were irradiated with a 635 nm laser (0.8 W cm^{-2}) for 5 min. The temperature change was recorded by a thermocouple and the photothermal images were acquired by an infrared thermal imager. In addition, the photothermal effect of the nanoparticles was investigated using different laser power intensities (0.2, 0.4, 0.6 and 0.8 w/cm^2) and the temperature change of different concentrations of NPs within 5 min under the same power of laser irradiation was monitored. To study the NPs' photostability, five cycles of laser on/off were carried out.

FITC-Labeled Fibrin Clots Assay.
Fluorescein isothiocyanate (FITC)-labeled fibrin clots were induced by the addition of 10 U mL^{-1} thrombin and 2.5 mM $CaCl_2$ into a fibrinogen solution containing 1 mg mL^{-1} fibrinogen (200 μL) and 1 mg mL^{-1} FITC-labeled fibrinogen (20 μL), followed by incubation at 37 °C for 1 h. The clot was incubated with Cu2Ir NPs (100 μg mL^{-1}, 600 μL) for 1 h and further irradiated with 635 nm laser (0.8 W cm^{-2}, 2 min) and evaluated through a CLSM.

In vitro hemolysis experiment.
A hemolysis assay was conducted to evaluate the hemocompatibility of Cu2Ir NPs. Firstly, the whole blood of a mouse treated with citrate was gathered and centrifuged at 3000 rpm for 3 min to obtain erythrocytes. Then, the erythrocytes were resuspended in an equal volume of PBS. PBS or water were used as negative and positive controls, respectively, which were added to 2 mL microtubes containing erythrocytes. Various concentrations (5, 10, 50, 100, 150 and 200 μg mL^{-1}) of Cu2Ir NPs were incorporated into erythrocytes for comparison with the controls. The mixture was then incubated in a 37 ºC water bath for 2 h, followed by centrifugation at 3000 rpm for 10 min. After centrifugation, 100 μL of supernatant from each sample was transferred to a 96-well plate and the absorbance was recorded with an enzyme marker at 540 nm. The equation for calculation of the hemolysis rate (%) is presented below.
Hemolysis rate (%) = $(A_0 - A_1) / (A_2 - A_1) \times 100\%$.

In vitro thrombus targeting of Cu2Ir NPs.
Fresh blood was collected and divided into tubes with equal volumes (5 mL). Each tube was mixed with thrombin (5 U μL^{-1}) and $CaCl_2$ (3 mM) to induce the formation of clots and cut into equal sizes. The artificial thrombus was then respectively incubated with PBS, Cu2Ir (no C(RGDfC)) or Cu2Ir NPs aqueous solution (0.1 mg mL^{-1}) to verify the targeting ability of the NPs. After incubation for 2 h, 4 h or 6 h, the thrombus clots were taken out and washed three times with PBS. The chemiluminescence of the thrombus clots was analyzed using an IVIS imaging system.

In vitro thrombolytic efficacy
The artificial thrombus was placed into a 5 mL glass vial, to which the mixture of 2.5 mL of PBS and 0.5 mL of different NPs solutions was added. The mixture was irradiated with a 635 nm laser. The weights of thrombus before and after thrombolytic treatment were measured to calculate the thrombolysis rate: thrombolysis rate = (weight before treatment – weight after treatment)/weight before treatment.

In Vitro Cell Cytotoxicity Test.
The human umbilical vein endothelial cells (HUVECs) were obtained from Peking Union Medical College Hospital (Peking, China). Fetal bovine serum (FBS), Ham's F12K, heparin, endothelial cell growth supplement (ECGS), L-glutamine, penicillin, and streptomycin were obtained from Corning (New York, USA). The cells were maintained in Ham's F12K medium with heparin (0.1 mg mL^{-1}), ECGS (0.05 mg mL^{-1}), 10% FBS, 1% L-glutamine and 1% penicillin/streptomycin at 37 °C containing 5% CO_2. For in vitro cytotoxicity tests, HUVECs were seeded into 96 well plates with a density of 1×10^4 per well. After 24 h, the cells were incubated with Cu2Ir NPs for another 24 h. The MTT assay was conducted following the standard protocol.

In Vitro Intracellular ROS Evaluation.
The reactive oxygen species (ROS) were imaged using specific agents, i.e., 2',7'-dichlorofluorescein diacetate (DCFH-DA) for the detection of ROS and dihydroethidium (DHE) for the detection of $O_2^{\bullet-}$. The cells were divided into eight groups: (i) control group; (ii) HUVEC cells incubated with H_2O_2 alone for 24 h; (iii-v) HUVEC cells were incubated with different NPs alone for 24 h; (vi-viii) HUVEC cells were incubated with different NPs (100 μg/mL) for 24 h followed by H_2O_2 treatment for 24 h. The cells were treated, or not treated, with H_2O_2 (200 μM). They were then washed with PBS three times, and then were incubated with DCFH-DA (10 μM) and DHE (10 μM) for 30 min at 37 °C. Subsequently, residual agents were removed using PBS. The images were obtained using a confocal microscope (LSM 800 Zeiss, Germany). Each experiment was performed independently three times.

Enzyme-Linked Immunosorbent Assay.

The supernatants were collected from the different treatment groups of RAW 264.7 cells, and the concentrations of the inflammatory factors, namely brain necrosis factor TNF-α and IL-6 were measured using an enzyme-linked immunosorbent assay (ELISA) kit. Each experiment was performed independently three times.

Construction of thrombus model

The male C57 BL/6J mice (8-10 weeks old) were anesthetized using isoflurane (2% of isoflurane, 0.5 mL min^{-1} oxygen) and the neck hair was shaved off. Then the skin around the neck was cut with surgical scissors, and the connective tissue and fat were peeled away to expose the carotid vessels. A 10% aqueous solution of $FeCl_3$-soaked filter paper (2 × 1 mm) was placed on the surface of the exposed carotid artery for 10 min, then the filter paper was removed. The vessel and surrounding tissue were washed with sterilized saline solution. An apparent aggregated embolus could be observed under the somatic microscope. The laser speckle imaging system (RWD, RFLSI III, Shenzhen, China) was used to monitor the hemodynamic changes before and after the induction of carotid artery thrombus using $FeCl_3$. All animal procedures were approved by the China Technology Industry Holdings (Shenzhen) Co., Ltd.

In vivo chemiluminescence imaging of thrombus

The C57 BL/6J mice (8-10 weeks old) with a thrombus model were intravenously administrated with PBS or Cu2Ir NPs (10 mg kg^{-1}), respectively. The mice were then anesthetized using isoflurane (2% of isoflurane, 0.5 mL min^{-1} oxygen), and placed on a heating platform at 37 °C. At predetermined times (0, 30, 60, 90, 120, 180, and 240 min) post NPs administration, the in vivo chemiluminescence imaging was carried out using an IVIS imaging system.

Evaluation of the thrombolytic effect of different formulations in vivo

To evaluate the therapeutic effect of different formulations on thrombi, the mice with carotid thrombosis or the lower extremity arterial thrombosis were randomly divided into three groups (n = 3 mice): PBS, free UK, Cu2Ir NPs groups. The different formulations were intravenously injected into the mice via the tail vein. For the NIR light-treated groups, the carotid arteries of the mice were illuminated with a 635 nm laser (0.8 W cm^{-2}, 5 min per session) at 60 min post NP administration. The temperature changes at the irradiation sites were monitored using an infrared thermal imaging system (Guide, Zhejiang, China). At different times (10, 20, 40, 60, 90, and 120 min) after the treatments, a laser speckle imaging system was used to observe the thrombosis sites, and applied to measure the blood flow velocity at different time points post treatments. Finally, the mice were euthanized, and the carotid arteries was excised for section and H&E staining. The areas and morphology of the thrombi after different treatments were observed under an optical microscope. The sections were analyzed using the Image J2x software (version 2.1.4.7) to the treatments, and thrombolytic efficiency was determined by the area ratio of vascular occlusion to total vascular lumen.

In vivo biosafety evaluation

To test the potential toxicity of Cu2Ir NPs, healthy male C57 BL/6J mice (8-10 weeks old) were intravenously injected with PBS or Cu2Ir NPs (10 mg kg^{-1}). One week after PBS or NPs administration, the mice were euthanized, and blood was withdrawn. Then the hematological parameters, including white blood cell count, red blood cell count, hemoglobin concentration, platelet count, monocyte, lymphocyte and neutrophil were analyzed using an automated hematology analyzer. The major organs (heart, liver, spleen, lung, and kidneys) were harvested for histological analysis. The tissues were fixed in 4% paraformaldehyde (PFA), followed by embedding in paraffin and sectioning at a thickness of 5 μm for H&E staining. The slices were observed under a digital microscope (Leica QWin).

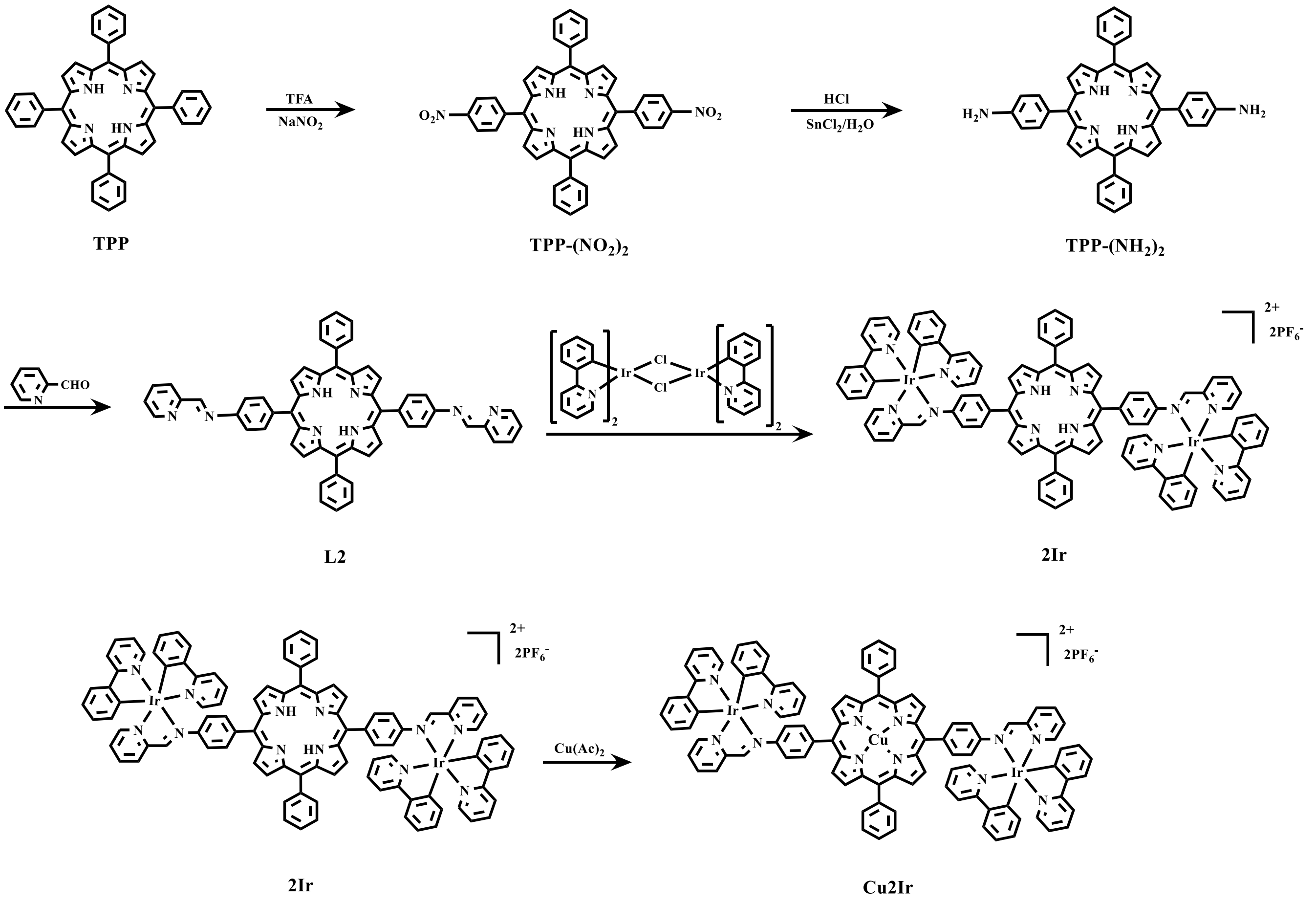

Scheme S1. Synthetic route for 2Ir and Cu2Ir.

Synthesis of TPP-(NO_2)$_2$ and TPP-(NH_2)$_2$ was based on literature reports.[3] **TPP-(NO_2)$_2$ ^{1}H NMR (600 MHz, $CDCl_3$)** δ 8.90 (d, J = 4.3 Hz, 2H), 8.87 (s, 2H), 8.75 (s, 2H), 8.72 (d, J = 4.2 Hz, 2H), 8.58 (d, J = 8.4 Hz, 4H), 8.34 (d, J = 8.4 Hz, 4H), 8.19 (d, J = 6.9 Hz, 4H), 7.76 (dt, J = 14.5, 7.3 Hz, 6H), -2.78 (s, 2H). **TPP-(NH_2)$_2$ ^{1}H NMR (600 MHz, DMSO-*d*$_6$)** δ 8.90 (s, 4H), 8.70 (s, 4H), 8.12 (d, J = 6.8 Hz, 4H), 7.82 (d, J = 7.9 Hz, 4H), 7.71 (d, J = 6.6 Hz, 6H), 6.95 (d, J = 7.9 Hz, 4H), 5.51 (s, 4H), -2.83 (s, 2H).

Synthesis of L2 TPP-(NH_2)$_2$ (0.193 g, 0.3 mmol) and 2-pyridinecarboxaldehyde (0.078 g, 0.72 mmol) were added into ethanol (60 mL), and the mixture was stirred at 78 °C for 8 h. After cooling to room temperature, the precipitate was filtered and recrystallized with ethanol to obtain the target compound with a yield of 80%. **^{1}H NMR (600 MHz, $CDCl_3$)** δ 8.96 (s, 2H), 8.95 (s, 2H), 8.93 (d, J = 4.1 Hz, 2H), 8.88 (d, J = 4.1 Hz, 2H), 8.86 (s, 2H), 8.79 (d, J = 4.7 Hz, 2H), 8.37 (d, J = 7.7 Hz, 2H), 8.28 (d, J = 7.9 Hz, 4H), 8.23 (d, J = 6.7 Hz, 4H), 7.88 (t, J = 7.6 Hz, 2H), 7.75 (dd, J = 12.2, 5.0 Hz, 6H), 7.70 (d, J = 7.9 Hz, 4H), 7.42 (dd, J = 7.0, 5.3 Hz, 2H), -2.73 (s, 2H).

Synthesis of 2Ir [Ir(ppy)$_2$Cl]$_2$ (0.107 g, 0.1 mmol) and auxiliary ligand L2 (0.0822 g, 0.1 mmol) were dissolved in a mixture of methanol (30 mL) and dichloromethane (30 mL) and refluxed for 6 h under the protection of N_2. After cooling to room temperature, excess KPF_6 was added to the mixture (to replace counterion Cl^-). After stirring for 0.5 h, filtering to remove the excess potassium salts, and removal of the solvent from the filtrate by rotary evaporation, the crude product was obtained. Finally, the purple solid product was purified by column chromatography on silica gel with dichloride and ethyl acetate as eluents (dichloromethane / acetone 10/3, v/v) with a yield of 85%. **^{1}H NMR (600 MHz, DMSO-*d*$_6$)** δ 10.05 (s, 1H), 8.94 (s, 1H), 8.88 (s, 1H), 8.83 (s, 1H), 8.74 (s, 1H), 8.64 (s, 1H), 8.53 (s, 1H), 8.39 (d, J = 7.4 Hz, 2H), 8.34 (d, J = 7.0 Hz, 1H), 8.23 (d, J = 10.4 Hz, 3H), 8.06 (s, 1H), 8.03 – 7.98 (m, 3H), 7.87 (d, J = 6.7 Hz, 6H), 7.83 (s, 1H), 7.45 (s, 1H), 7.38 (s, 1H), 7.22 (d, J = 8.1 Hz, 2H), 7.09 – 7.03 (m, 2H), 6.96 (dd, J = 14.6, 7.2 Hz, 2H), 6.33 (d, J = 7.3 Hz, 1H), 6.31 (d, J = 7.1 Hz, 1H), -3.02 (s, 1H). **^{13}C NMR (600 MHz, DMSO-*d*$_6$)** δ 170.58 (s), 167.55 (s), 167.11 (s), 156.05 (s), 151.56 (s), 151.03 – 150.87 (m), 150.64 (s), 150.02 (s), 149.66 – 149.50 (m), 147.67 (s), 144.47 (s), 144.34 (s), 141.58 (s), 141.03 (s), 140.40 (s), 139.60 (s), 139.44 (s), 134.64 (s), 134.21 (s), 131.92 (s), 131.71 (s), 131.09 – 131.01 (m), 130.72 (s), 130.33 (s), 128.78 – 128.61 (m), 127.56 (s), 125.67 (s), 125.13 (s), 124.75 (s), 124.60 – 124.52 (m), 123.01 (s), 122.64 – 122.56 (m), 121.35 – 121.27 (m), 120.90 (s), 120.75 (s), 120.16 (s), 119.02 (s). Mass spectrometry (ESI-MS, m/z): $[C_{100}H_{70}Ir_2N_{12}]^{2+}$ calcd. 912.255; found: 912.2762.

Synthesis of Cu2Ir 2Ir (0.1 mmol, 0.1824 g) and copper acetate (0.4 mmol, 0.080 g) were added to a 100 mL beaker with DMF as the reaction solvent, and the reaction was stirred at 77 °C for 8 h. The product was extracted with dichloromethane and water, and the solvent in the system was removed by rotary evaporator, and the red solid product, i.e., the bimetallic iridium complex coupling Cu2Ir,

was dried. The product is a bimetallic iridium complex coupling compound Cu2Ir, with a yield of 86%. Mass spectrometry (ESI-MS, m/z): $[C_{100}H_{68}CuIr_2N_{12}]^{2+}$ calcd. 942.71; found: 942.8454.

NMR and MS spectra of compounds

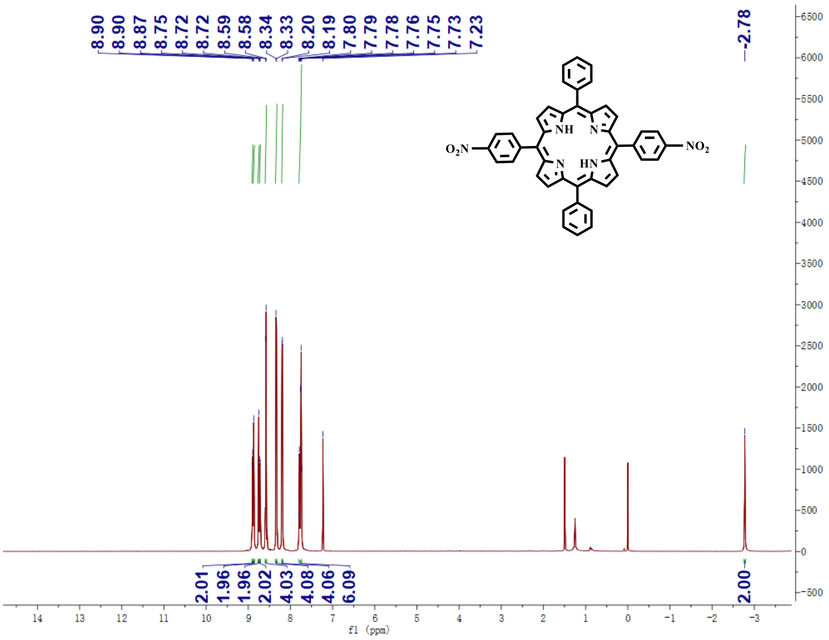

Figure S1 ^{1}H NMR spectrum of TPP-$(NO_2)_2$ in $CDCl_3$ at room temperature.

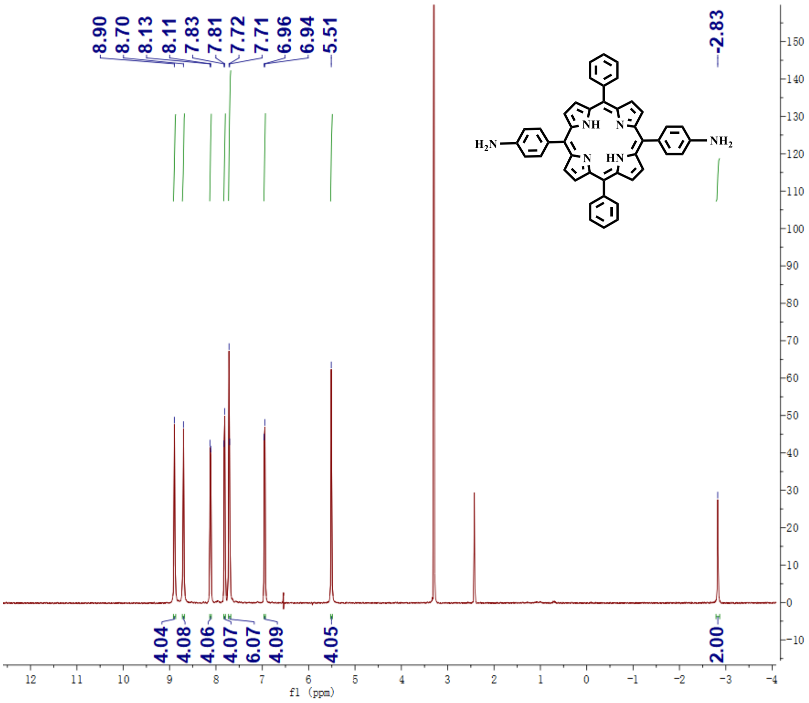

Figure S2 ^{1}H NMR spectrum of TPP-$(NH_2)_2$ in DMSO-d_6 at room temperature.

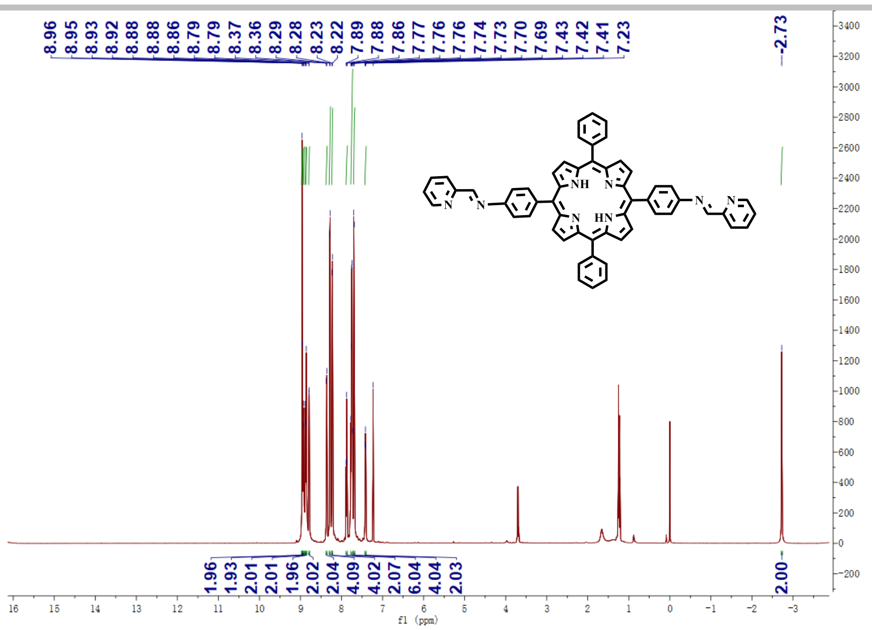

Figure S3 ^{1}H NMR spectrum of L2 in $CDCl_3$ at room temperature.

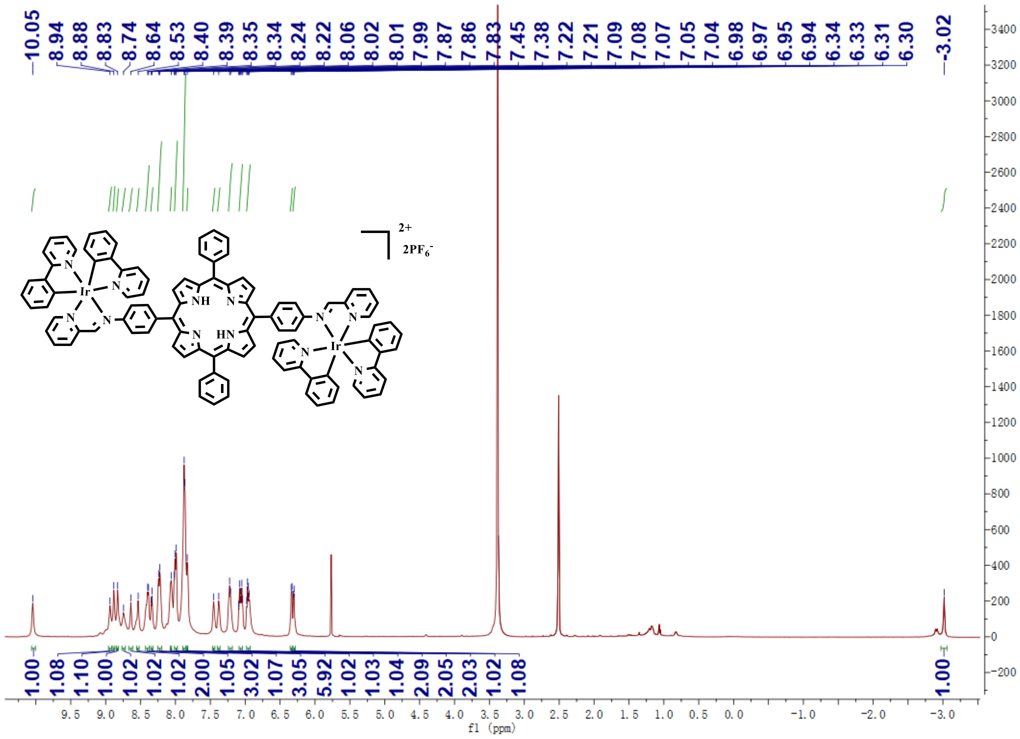

Figure S4 ^{1}H NMR spectrum of 2Ir in DMSO-d_6 at room temperature.

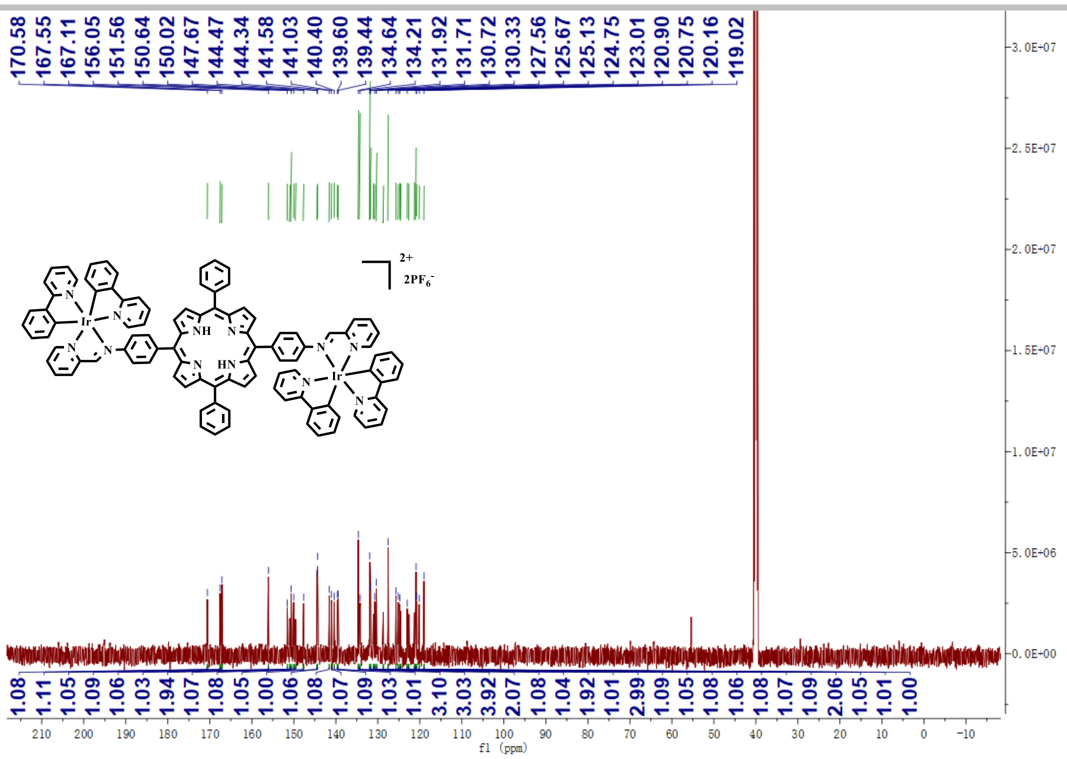

Figure S5 ^{13}C NMR spectrum of 2Ir in DMSO-d_6 at room temperature.

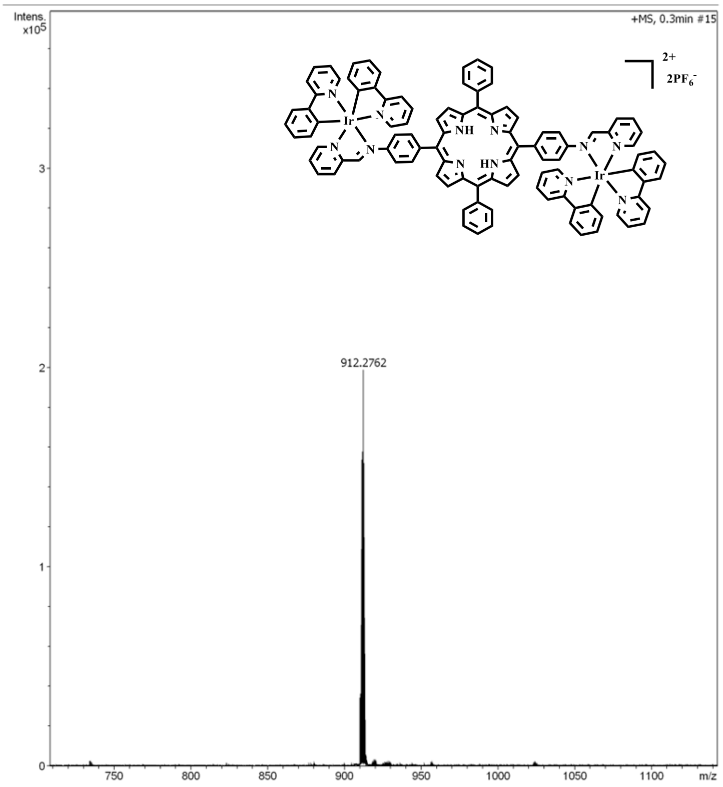

Figure S6 Mass spectrum of 2Ir at room temperature.

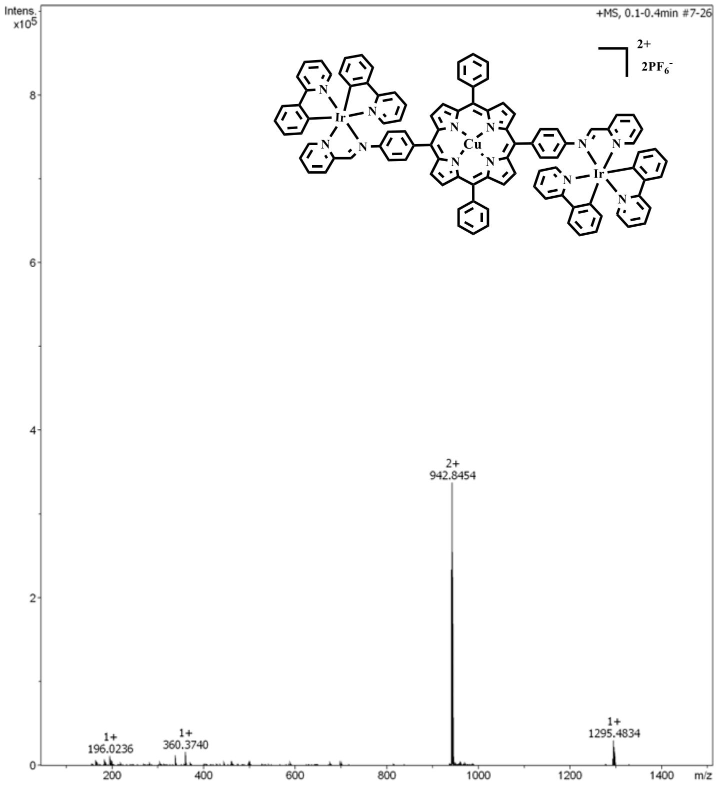

Figure S7 Mass spectrum of Cu2Ir at room temperature.

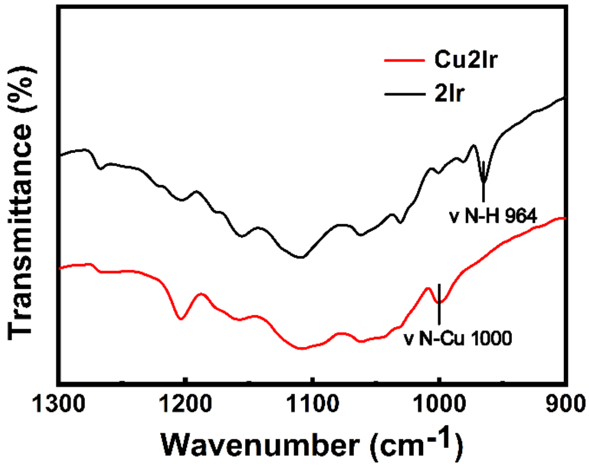

Figure S8. FT-IR spectra of 2Ir and Cu2Ir.

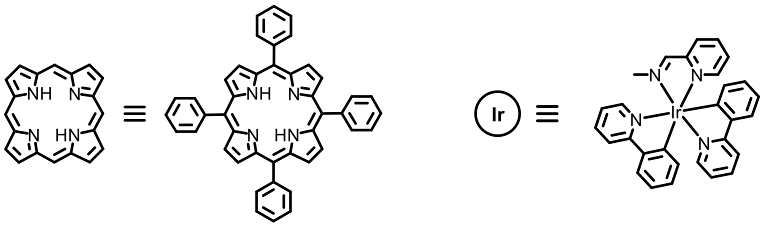

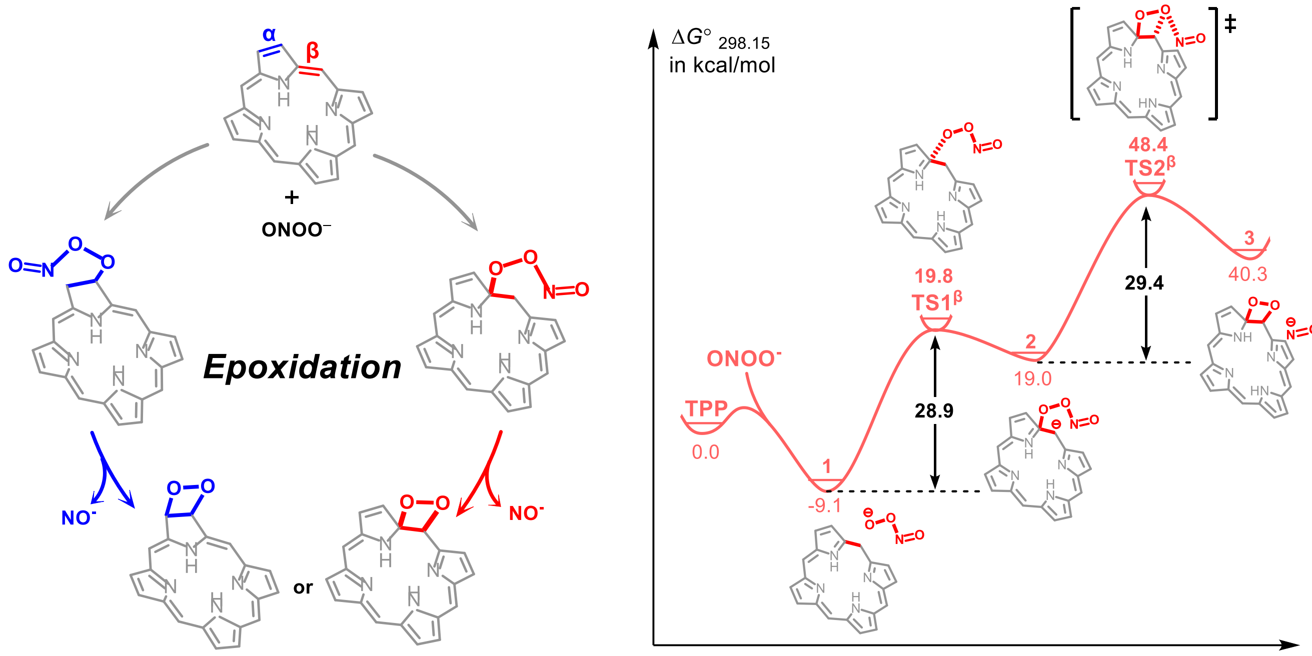

Figure S9 The mechanistic diagram of $ONOO^-$ reacting at different sites of TPP and Gibbs energy changes (in kcal/mol) in the formation of an unstable dioxetane by $ONOO^-$ oxidizing TPP at the β site. (TS = Transition State).

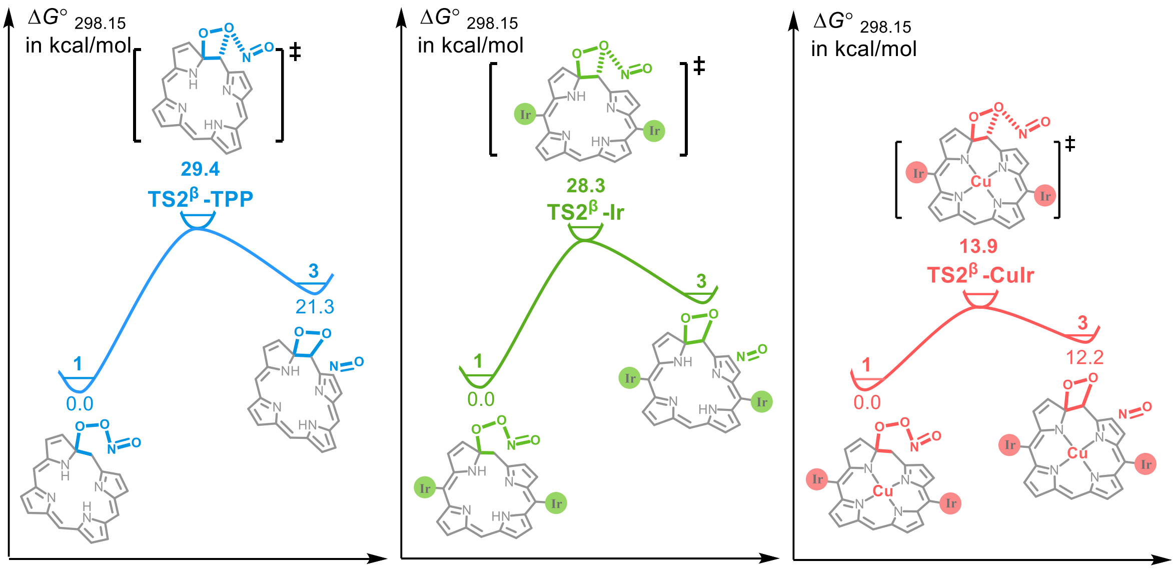

Figure S10 Gibbs energy changes (in kcal/mol) of the rate-determining step in the formation of unstable intermediates by $ONOO^-$ oxidizing TPP, 2Ir and Cu2Ir.

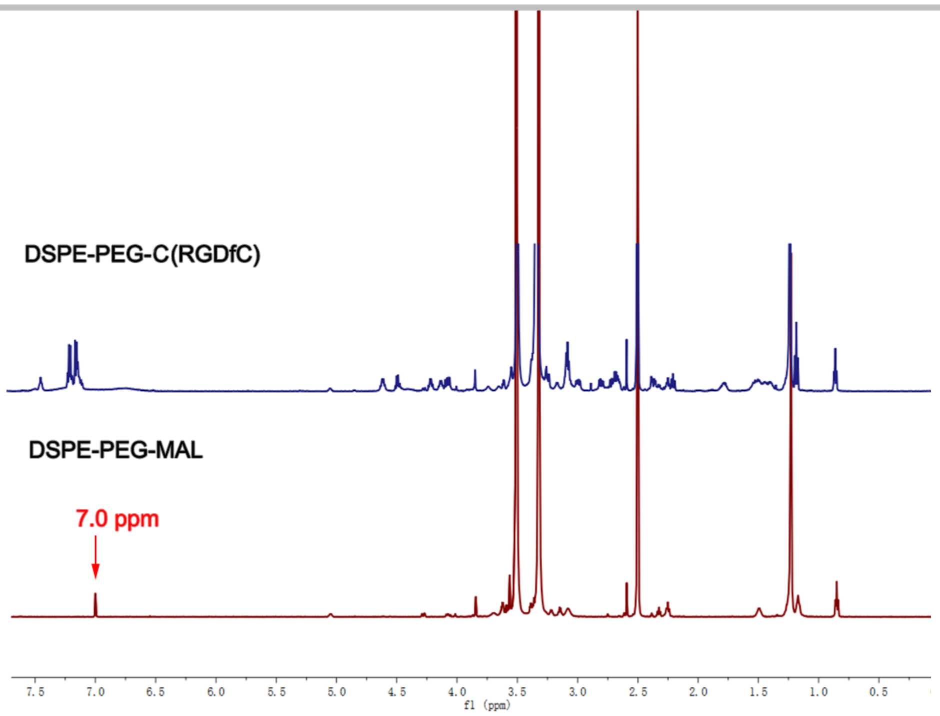

Figure S11 ^{1}H NMR spectra of DSPE-PEG-MAL and DSPE-PEG-C(RGDfC) (600 MHz, DMSO-d_6). The disappearance of the maleimide group signal at 7.0 ppm indicated the successful conjugation of C(RGDfC) to DSPE-PEG-MAL.

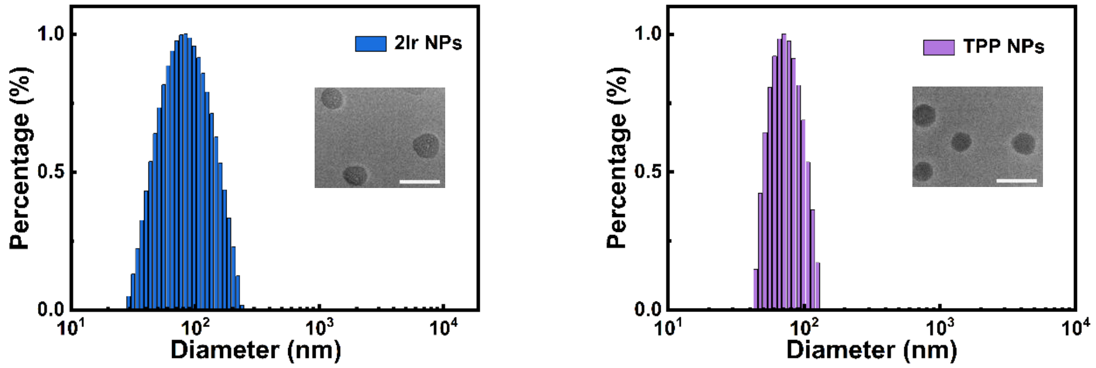

Figure S12 Size and TEM image of 2Ir NPs and TPP NPs. Scale bar = 200 nm.

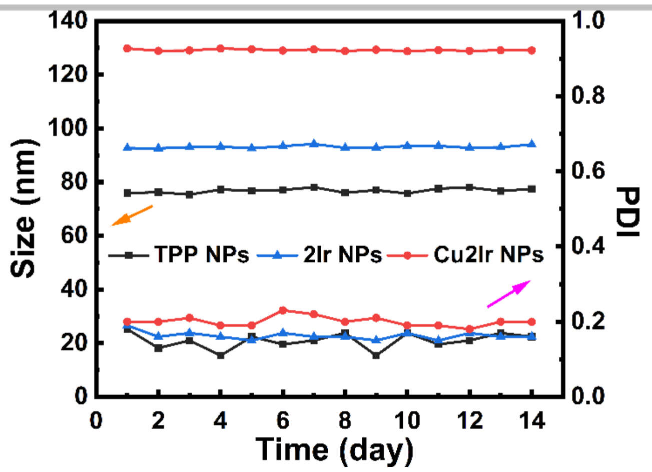

Figure S13 Stability of size distribution changes over 14 days for TPP NPs, 2Ir NPs and Cu2Ir NPs.

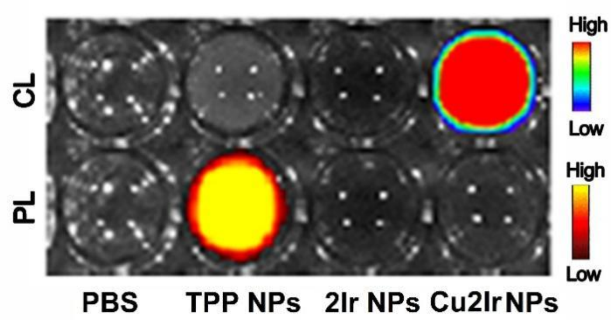

Figure S14 CL and PL imaging of PBS, TPP NPs, 2Ir NPs and Cu2Ir NPs.

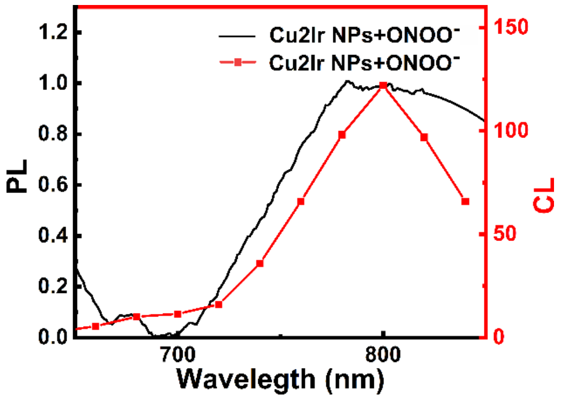

Figure S15 CL and PL spectra of the intermediates of Cu2Ir NPs reacted with $ONOO^{-}$.

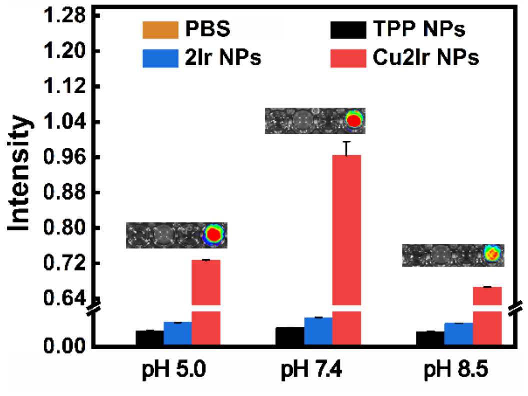

Figure S16 Normalised chemiluminescence plots of PBS, TPP NPs, 2Ir NPs and Cu2Ir NPs at different pH values.

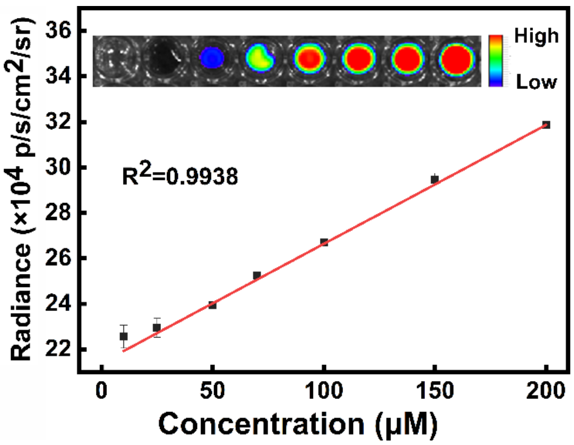

Figure S17 Correlation of persistent luminescence intensity of Cu2Ir NPs versus $ONOO^-$ concentration. Inset: the corresponding chemiluminescence acquired on an IVIS imaging system.

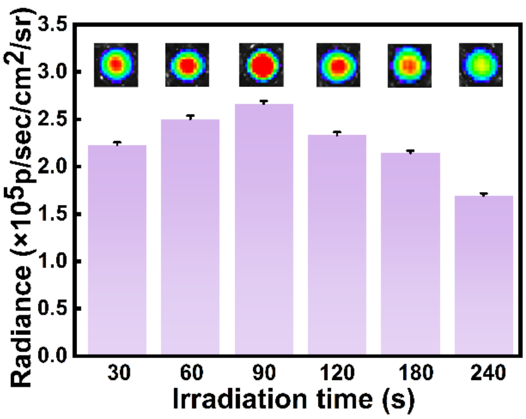

Figure S18 The quantification of corresponding persistent luminescence intensities under different times of laser irradiation.

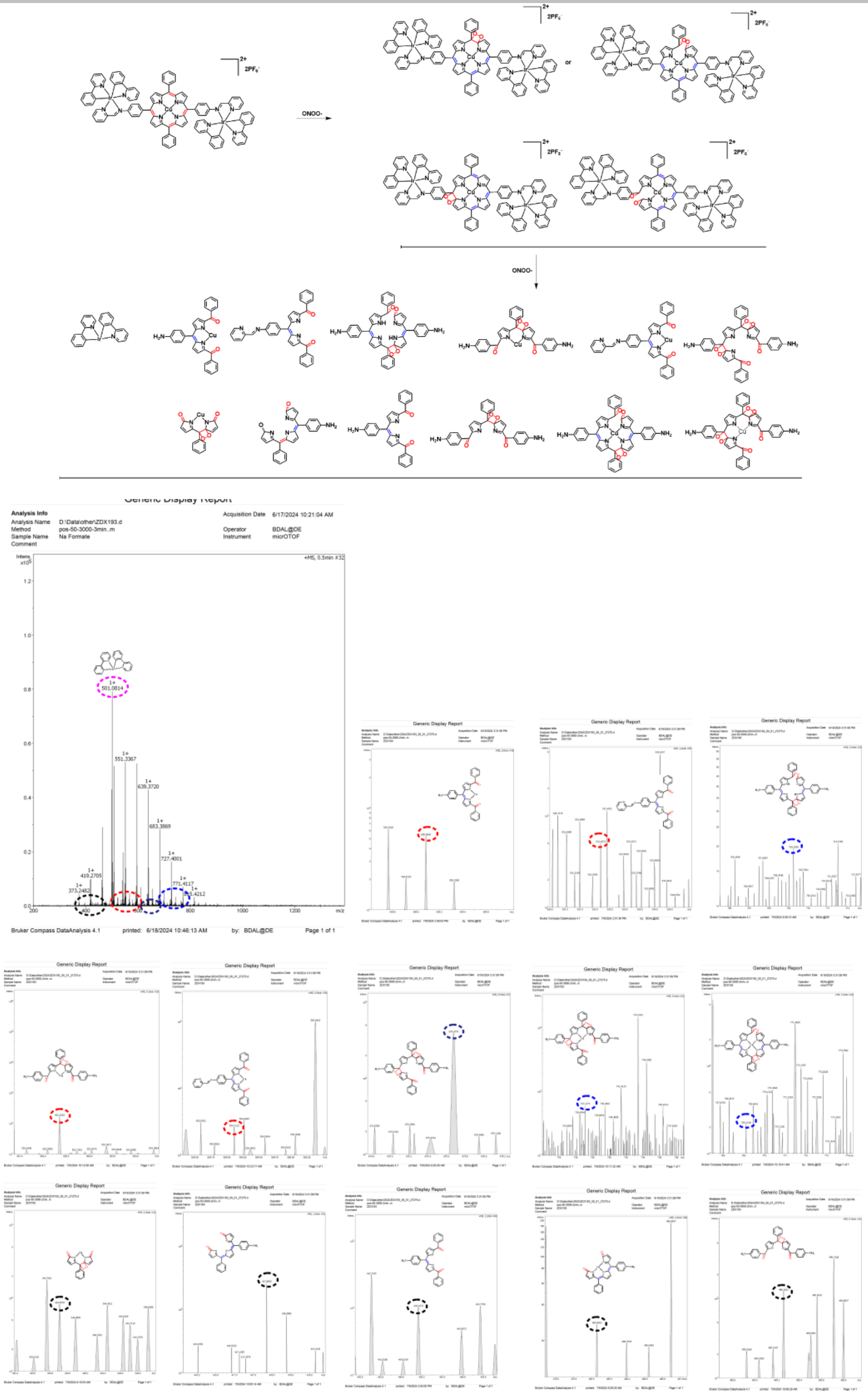

Figure S19 The proposed chemiluminescence reaction mechanism of Cu2Ir and ONOO$^-$ and the related mass spectra.

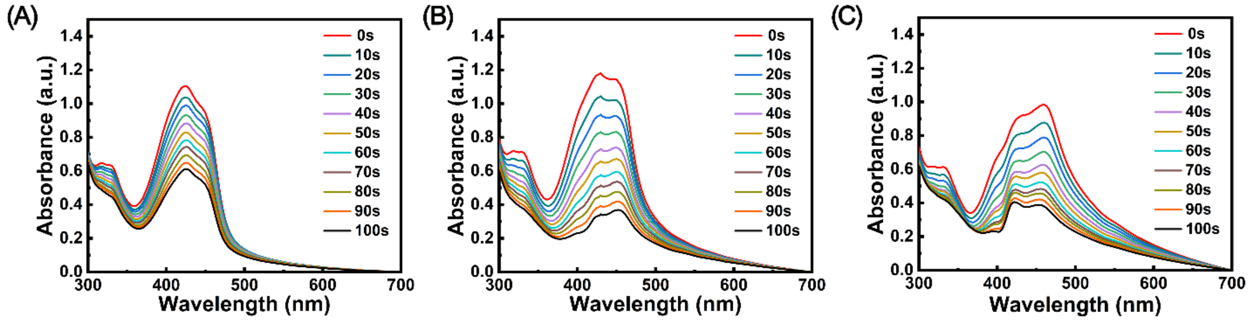

Figure S20 UV–vis absorption spectral changes of DPBF (1.5×10^{-5} M) in the presence of TPP NPs (A), 2Ir NPs (B) and Cu2Ir NPs (C) upon exposure to light (635 nm, 0.8 w/cm^2) during 100 s.

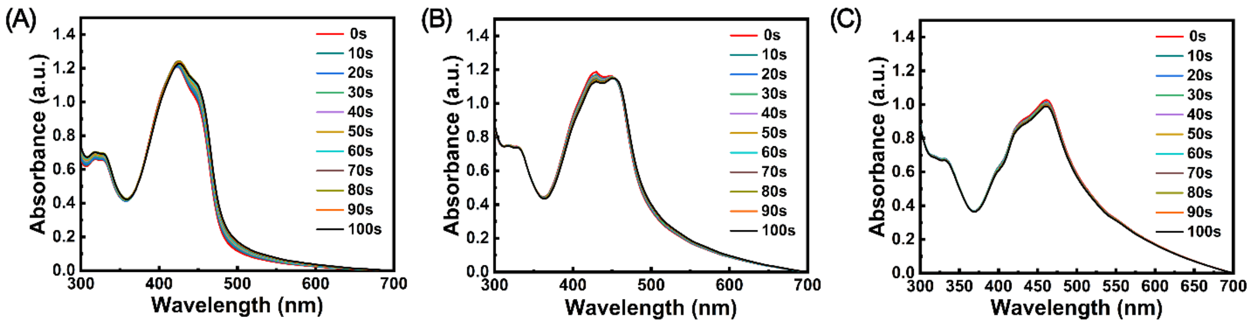

Figure S21 UV–vis absorption spectral changes of DPBF (1.5×10^{-5} M) in the presence of TPP NPs (A), 2Ir NPs (B) and Cu2Ir NPs (C) during 100 s.

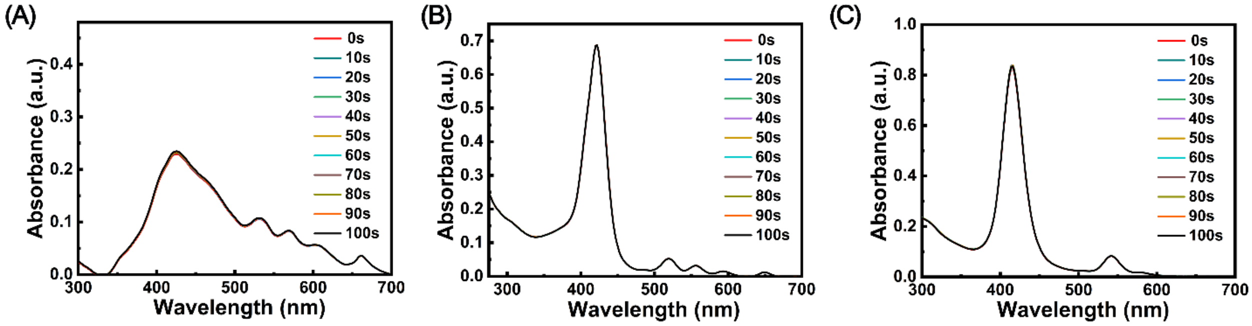

Figure S22 UV–vis absorption spectral changes of TPP NPs (A), 2Ir NPs (B) and Cu2Ir NPs (C) upon exposure to light (635 nm, 0.8 w/cm^2) during 100 s.

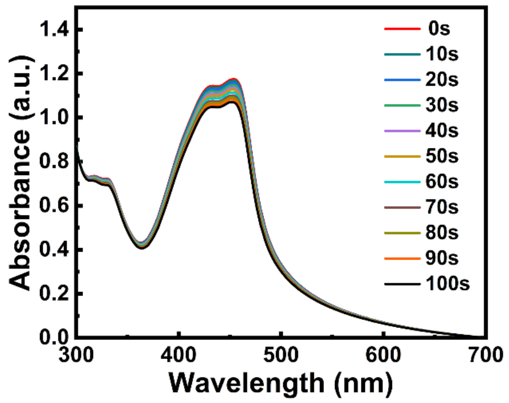

Figure S23 UV–vis absorption spectral changes of DPBF (1.5×10^{-5} M) upon exposure to light (635 nm, 0.8 w/cm^2) during 100 s.

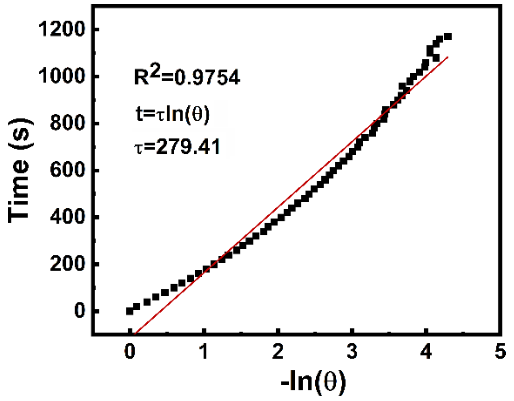

Figure S24 Plot of cooling time versus negative natural logarithm of the temperature obtained from the cooling stage.

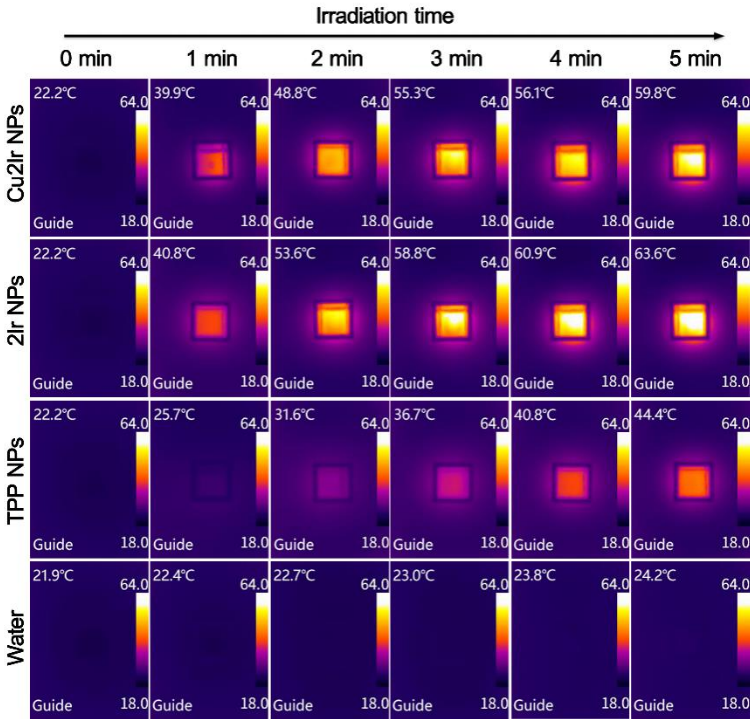

Figure S25 Infrared images of NPs under laser irradiation.

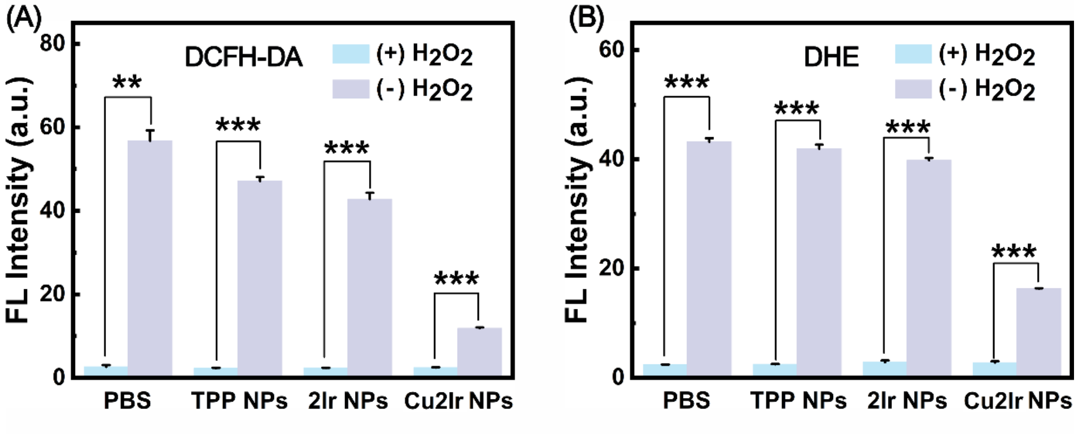

Figure S26 Quantitative analysis of the intracellular fluorescence intensity of NPs scavenged RONS and $O_2^{\bullet -}$ activity induced by H_2O_2-treated HUVECs in vitro.

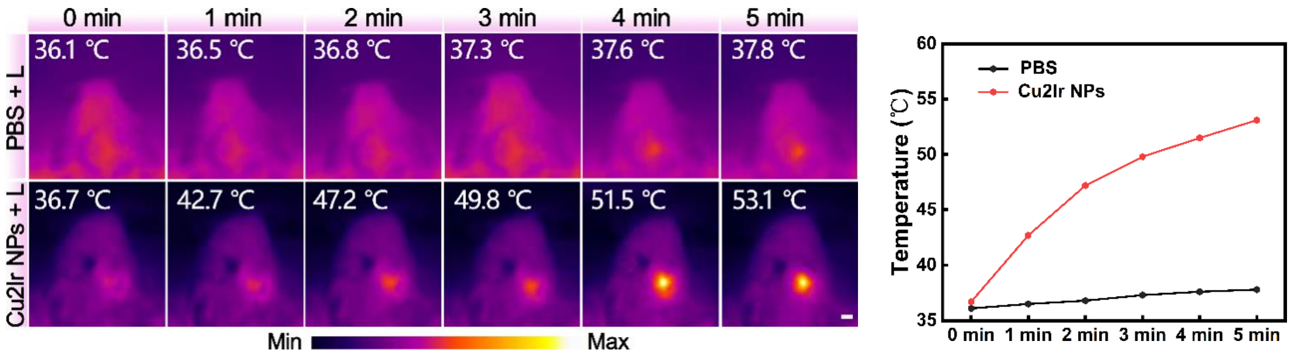

Figure S27 Thermal images of the thrombus site after 635 nm laser irradiation (0.8 W 0.8w/cm^2) for different time. Scale bar = 3 mm. Photothermal profiles of the thrombus after injecting PBS or Cu2Ir NPs under 635 nm laser irradiation.

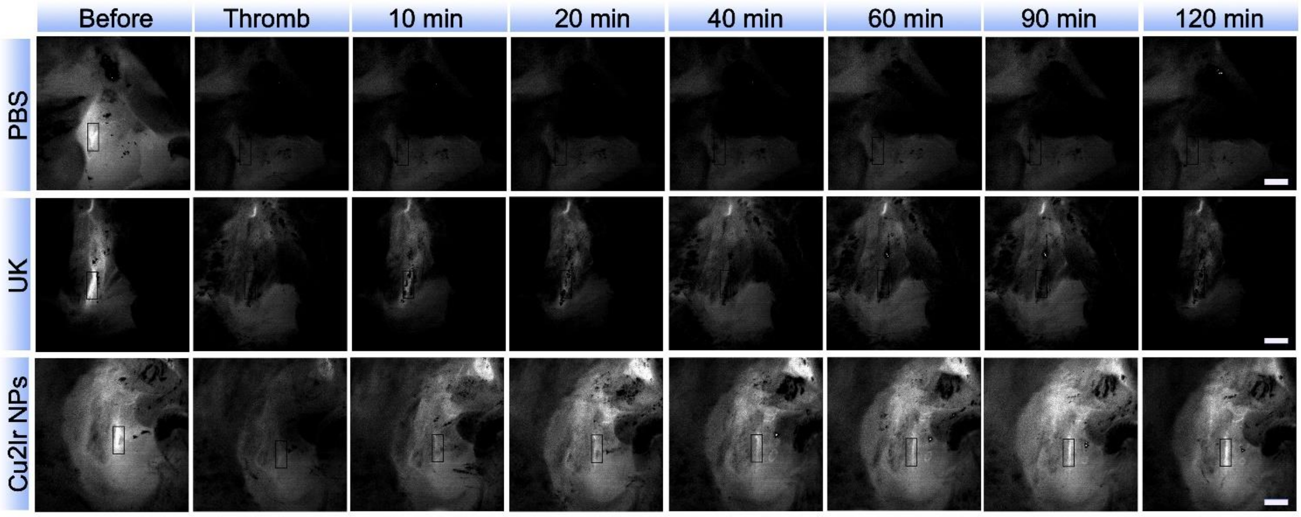

Figure S28 Representative LSBFMS images of the mice carotid artery after $FeCl_3$ induction and different therapeutic treatments, including PBS, free UK and Cu2Ir NPs. Scale bars = 5 mm.

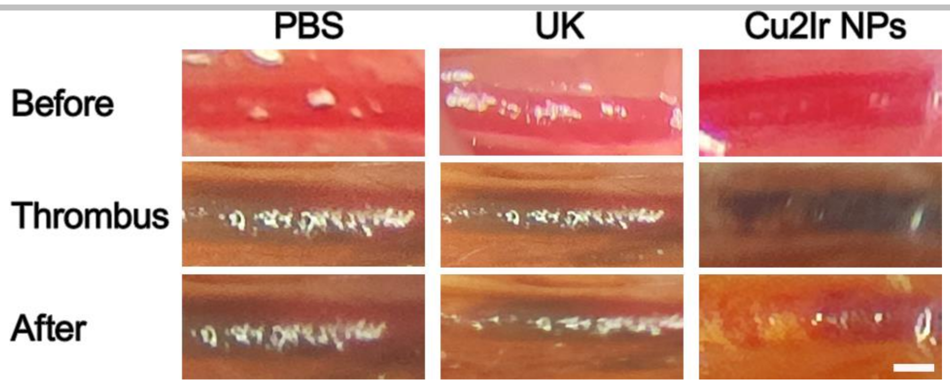

Figure S29 Representative thrombus pictures before and after different treatments. Scale bar = 1 mm. Data are shown as means ± SD (n = 3).

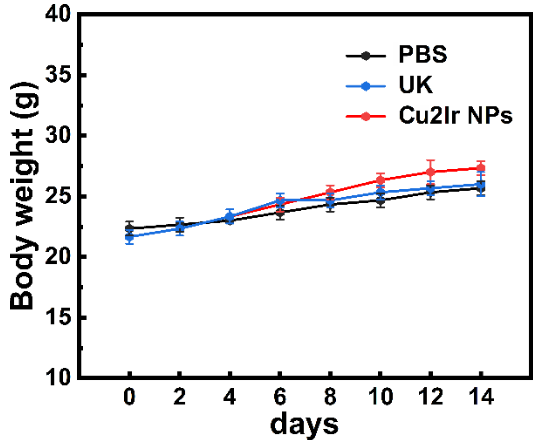

Figure S30 Body weight variations of thrombolytic mice after various treatments.

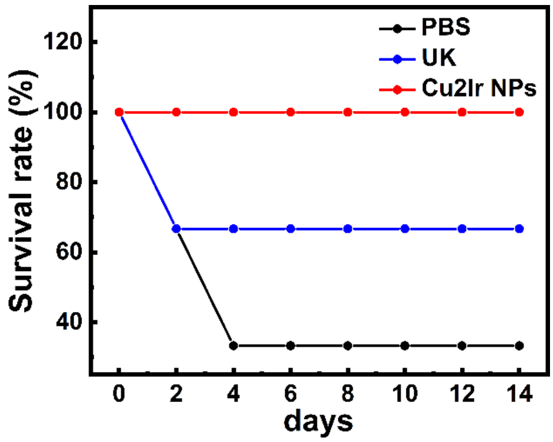

Figure S31 Survival rate for different groups of mice.

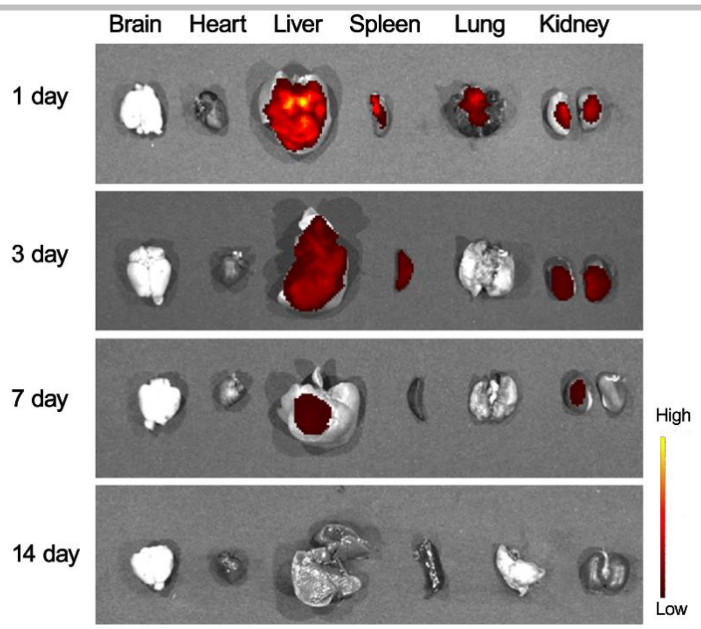

Figure S32 Images of excised tissue after loading IR780 fluorescence with the Cu2Ir NPs. The results are presented as Mean ± SD (n = 3).

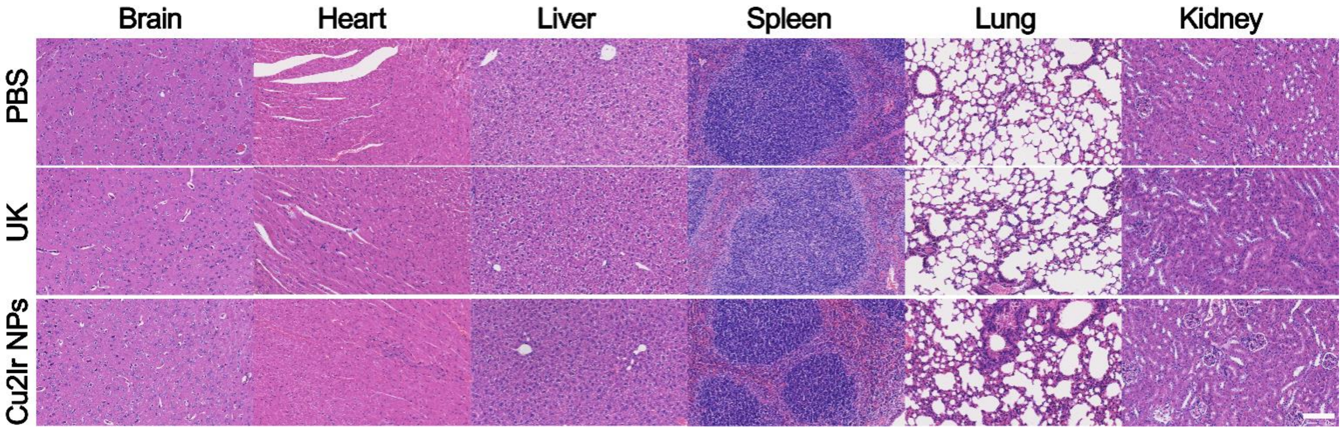

Figure S33 H&E staining of tissue sections from major organs of mice which were euthanized at 14-days after injection with PBS, free UK and Cu2Ir NPs. Scale bar = 100 μm.

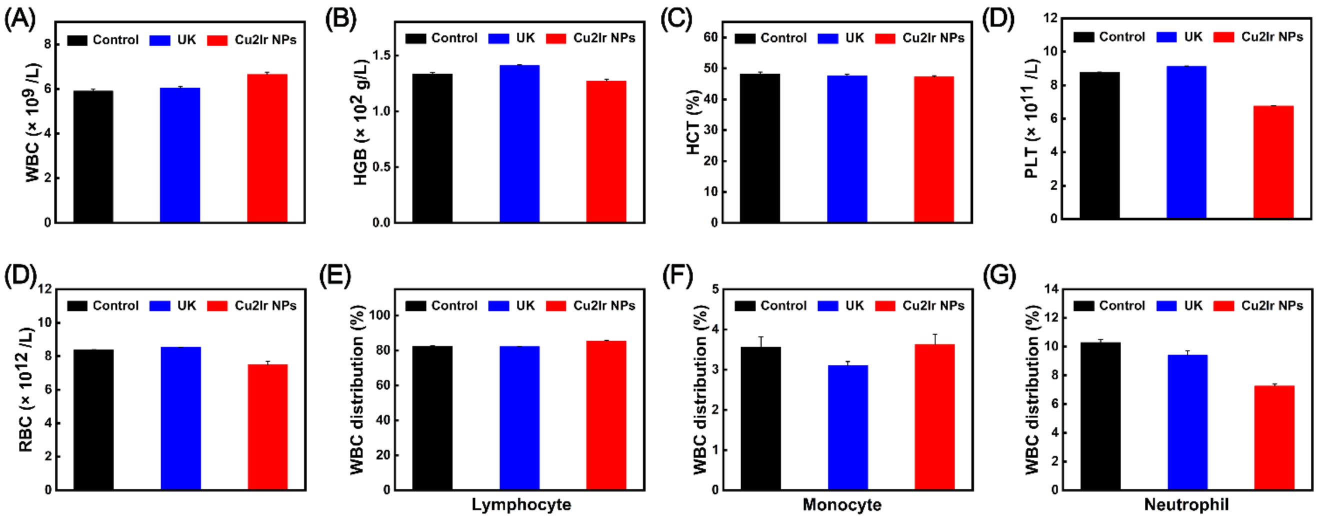

Figure S34 The routine blood test data of the mice with different treatments. Data are presented as mean ± SD (n = 3 mice). WBC is white bold cell; HGB is hemoglobin; HCT is hematocrit; PLT is platelet count; RBC is red blood cell.

1. C. Chen, H. Gao, H. Ou, R. T. K. Kwok, Y. Tang, D. Zheng, D. Ding, *J. Am. Chem. Soc*. **2022**, *144*, 3429-3441.
2. X. Liu, B. Li, F. Fu, K. Xu, R. Zou, Q. Wang, B. Zhang, Z. Chen, J. Hu, *Dalton Trans*. **2014**, *43*, 11709-11715.
3. R. Luguya, L. Jaquinod, F. R. Fronczek, *Tetrahedron* **2004**, *60*, 2757-2763.
